# Biomarkers Associated with Mortality in Aortic Stenosis: A Systematic Review and Meta-Analysis

**DOI:** 10.3390/medsci9020029

**Published:** 2021-05-17

**Authors:** Madeline White, Ranu Baral, Alisdair Ryding, Vasiliki Tsampasian, Thuwarahan Ravindrarajah, Pankaj Garg, Konstantinos C. Koskinas, Allan Clark, Vassilios S. Vassiliou

**Affiliations:** 1 Norwich Medical School, University of East Anglia, Norwich Research Park, Norwich NR4 7UQ, UK; maddie.white@uea.ac.uk (M.W.); ranubaral@hotmail.com (R.B.); alisdair.ryding@nnuh.nhs.uk (A.R.); t.ravindrarajah@uea.ac.uk (T.R.); p.garg@uea.ac.uk (P.G.); allan.clark@uea.ac.uk (A.C.); v.vassiliou@uea.ac.uk (V.S.V.); 2 Norfolk and Norwich University Hospital, Norwich NR4 7UQ, UK; 3 Department of Cardiology, Bern University Hospital, Freiburgstrasse 18, 3010 Bern, Switzerland; kckoskinas@gmail.com

**Keywords:** aortic valve stenosis, biomarkers, mortality, meta-analysis

## Abstract

The optimal timing of aortic valve replacement (AVR) remains controversial. Several biomarkers reflect the underlying pathophysiological processes in aortic stenosis (AS) and may be of use as mortality predictors. The aim of this systematic review and meta-analysis is to evaluate the blood biomarkers utilised in AS and assess whether they associate with mortality. PubMed and Embase were searched for studies reporting baseline biomarker level and mortality outcomes in patients with AS. A total of 83 studies met the inclusion criteria and were systematically reviewed. Of these, 21 reporting brain natriuretic peptide (BNP), N-terminal pro B-type natriuretic peptide (NT-proBNP), Troponin and Galectin-3 were meta-analysed. Pooled analysis demonstrated that all-cause mortality was significantly associated with elevated baseline levels of BNP (HR 2.59; 95% CI 1.95–3.44; *p* < 0.00001), NT-proBNP (HR 1.73; 95% CI 1.45–2.06; *p* = 0.00001), Troponin (HR 1.65; 95% CI 1.31–2.07; *p* < 0.0001) and Galectin-3 (HR 1.82; 95% CI 1.27–2.61; *p* < 0.001) compared to lower baseline biomarker levels. Elevated levels of baseline BNP, NT-proBNP, Troponin and Galectin-3 were associated with increased all-cause mortality in a population of patients with AS. Therefore, a change in biomarker level could be considered to refine optimal timing of intervention. The results of this meta-analysis highlight the importance of biomarkers in risk stratification of AS, regardless of symptom status.

## 1. Introduction

Aortic stenosis (AS) is the most common valvular heart disease in the elderly in Western countries [1,2], with an estimated prevalence of 5–7% in the general population over 65 years [3]. According to the current guidelines, intervention is recommended for patients with symptomatic or rapidly progressing severe aortic stenosis and for asymptomatic patients with significant decline of the left ventricular (LV) ejection fraction (EF) < 50% [4]. However, there are increasing concerns that, by the time of symptom development or decline of LV function, irreversible myocardial damage has already occurred [5].

The use of blood biomarkers is one of the simple and most important emerging methods of risk stratification of patients with aortic stenosis that has drawn a great deal of research interest. A significant increase in blood biomarkers can reflect early decompensation of the disease process, a finding that can be hugely advantageous for decision-making surrounding the optimal timing for intervention. Additionally, increased levels of certain biomarkers have been associated with adverse prognosis and increased mortality [6,7,8]. This can be an extremely useful clue in the management of patients with AS, especially useful for those who do not meet conventional criteria for intervention, namely the cohort of patients with asymptomatic severe AS.

Despite the availability of numerous publications, including two previous systematic reviews [9,10], to our knowledge there are none to date which have meta-analysed multiple biomarkers in AS. Therefore, the purpose of this work was to summarise the evidence on which biomarkers effectively predict mortality in patients with AS. We believe this is important as it may contribute to the implementation of biomarker use in future practice to improve risk stratification and identification of patients who would benefit from early intervention.

## 2. Methods

### 2.1. Search Strategy

This systematic review was conducted in accordance with the Preferred Reporting Items for Systematic Reviews and Meta-Analyses (PRISMA) statement [11] and was registered with PROSPERO (ID CRD42020170179). PubMed and Embase were searched using key terms including ‘aortic stenosis’, ‘blood biomarkers’, ‘BNP’, ‘troponin’, ‘ST2’ and ‘galectin’. The full search strategy is available in Supplementary Material, Figure S1. The search was conducted from January 1965 through to November 2019 to reflect both historical and contemporary practice. Reference lists from yielded studies were scrutinised for additional relevant citations.

### 2.2. Eligibility Criteria

Studies were included if they had mortality data on adults (>18 years) diagnosed with at least mild AS with known baseline blood biomarker levels prior to any medical or surgical intervention. Any study design was eligible excluding narrative reviews, editorials, case reports and case series. Non-English studies were excluded. If duplication occurred, the study with the largest sample size overall was included.

### 2.3. Data Extraction

Titles and abstracts were screened by two independent reviewers (MW and RB). Any articles identified as potentially relevant underwent full-text appraisal for inclusion using the piloted eligibility criteria. Any studies that were not eligible were removed and the reasons behind this judgment were recorded in the PRISMA diagram (Supplementary Material, Figure S2). Relevant information such as identification of study, participant information, baseline biomarker measure, interventions and mortality data were extracted. Verification checks were completed against those reported in the study to ensure data accuracy.

### 2.4. Statistical Analysis

Studies were grouped according to baseline biomarker and effect estimates were directly extracted. Where this was not reported, the raw data was extracted to calculate risk ratio (RR). Due to the substantial clinical heterogeneity between studies, an inverse-variance random-effects model for meta-analysis was used, which has been shown to be advantageous even if statistical heterogeneity is low [12]. This was done using Review Manager (RevMan) 5.3 software [13]. Statistical significance was set at *p* < 0.05.

Statistical heterogeneity between pooled studies was assessed using the I^2^ statistic. Heterogeneity was considered substantial when I^2^ was greater than 50% [14]. To identify the extent that each study contributed to the pooled effect estimate, sensitivity analyses were conducted by excluding one study at a time and recalculating the pooled estimate. Publication bias was determined graphically by assessing asymmetry of funnel plots.

### 2.5. Quality Analysis

The Newcastle-Ottawa Scale (NOS), a nine-point scale was used to assess the methodological quality and internal validity of included cohort and case-control studies. Eligible papers underwent meta-analysis irrespective of quality to utilise all available data.

## 3. Results

### 3.1. Identification and Selection of Studies

The literature search yielded 2886 studies from PubMed and Embase as shown in the PRISMA diagram (Supplementary Material, Figure S2). Initially 2785 studies were excluded after duplicates were removed and studies were assessed for eligibility at the title and abstract level. A total of 243 studies underwent full-text evaluation and 83 of those studies met the inclusion criteria and were included in the systematic review. The main exclusion criteria were not reporting baseline biomarker levels and/or mortality outcomes.

### 3.2. Natriuretic Peptides

Brain natriuretic peptide (BNP) and its prohormone N-terminal pro B-type natriuretic peptide (NT-proBNP) reflect ventricular or atrial cardiomyocyte stretch [15]. A total of 26 eligible studies with 7057 participants reported BNP and 33 studies with 8597 participants reported NT-proBNP (Supplementary Material, Tables S1 and S2). Most of these studies were large and observational, with a follow-up period of over one year. The BNP population had a mean age of 61 years and 42% were male, whereas the mean age of participants in the NT-proBNP population was higher at 78 years and 57% were male. All-cause mortality was reported in 21 of the BNP studies [6,7,8,16,17,18,19,20,21,22,23,24,25,26,27,28,29,30,31,32,33] and the remaining studies reported major adverse cardiovascular events (MACE) [34,35,36,37,38]. The vast majority found that BNP had a significant association with all-cause mortality, with an average threefold increase in death on increasing levels of BNP. This significance tended to remain in multivariate analysis after adjustment for various clinical factors. Importantly, only one study found that BNP was not significantly associated with all-cause mortality [26]. Similarly, 23 of the NT-proBNP studies reported all-cause mortality [6,17,22,39,40,41,42,43,44,45,46,47,48,49,50,51,52,53,54,55,56,57,58] and the other 10 studies reported composite mortality outcomes such as MACE [59,60,61,62,63,64,65,66,67,68]. Again, only one study found that NT-proBNP was not significantly associated with all-cause mortality [52]. On multivariate analysis, the majority found that the significance remained after adjustment of other variables. When reviewing the studies for MACE, a similar pattern was observed for both BNP and NT-proBNP.

**Figure 1:**
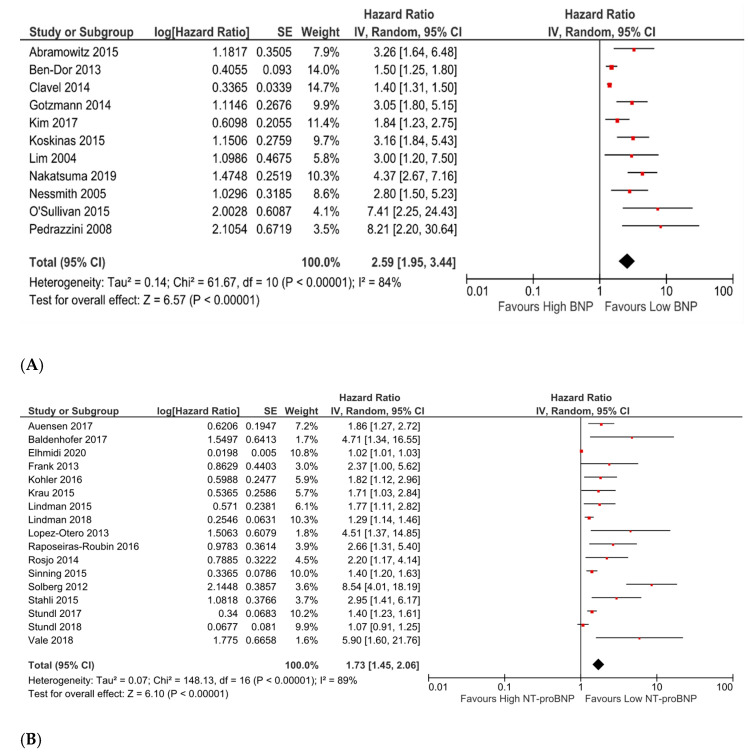
(**A**). Forest plot of hazard and risk ratios of all-cause mortality for high vs. low levels of baseline BNP using a random-effect model. This indicates that when comparing groups of high vs. low BNP as defined by the authors, there was over double the risk of mortality in the higher group. CI: confidence interval; IV: inverse variance. (**B**). Forest plot of hazard and risk ratios of all-cause mortality for high vs. low levels of baseline NT-proBNP using a random-effect model. This indicates that there was 1.73 times the risk of mortality associated with the high-level group. CI: confidence interval; IV: inverse variance.

Among the studies systematically reviewed, eleven studies analysing BNP and seventeen studies analysing NT-proBNP reported an effect size for all-cause mortality in patients with high vs. low levels of baseline biomarker. From these studies, the HRs and RRs were obtained for meta-analysis. Pooled analyses demonstrated a statistically significant increase in all-cause mortality for high vs. low levels of both baseline BNP (pooled HR 2.59; 95% CI 1.95 to 3.44; *p* < 0.00001; Figure 1A) and baseline NT-proBNP (pooled HR 1.73; 95% CI 1.45 to 2.06; *p* < 0.00001; Figure 1B).

The majority of studies included in the meta-analyses for both natriuretic peptides were found to be of at least fair quality using the Newcastle Ottawa Scale (NOS) (Supplementary Material, Table S3). Both meta-analyses produced an I^2^ statistic over 50% suggesting high risk of heterogeneity. Sensitivity analysis by excluding any single study from the analysis and recalculating the pooled effect did not substantially change the result of either meta-analysis. To assess the presence of publication bias, funnel plots were assessed with some asymmetry noted (Supplementary Material, Figure S3).

### 3.3. Troponin

Cardiac troponins such as Troponin I and Troponin T are cardiac regulatory proteins believed to be elevated in the presence of cardiomyocyte necrosis [10]. A total of 18 studies analysing Troponin were systematically reviewed, which yielded 5993 participants. Most studies were large and observational with only two of the studies having a follow-up of under one year. Participants had an average age of 80 years and 57% were male (Supplementary Material, Table S4). Of the included studies, 15 studies reported all-cause mortality [8,16,40,42,43,48,50,54,55,58,69,70,71,72,73]. Almost all studies found a significant association between Troponin level and mortality, with studies showing an average twofold increase in risk of death for higher Troponin, which persisted after multivariate adjustment. Importantly, three studies found that Troponin was not significantly associated with all-cause mortality [16,50,54]. Among the studies that reported MACE, all found a significant association between Troponin level and MACE [60,64,74], with one study describing a 10-fold risk of MACE in those with Troponin >10 ng/L [64].

**Figure 2:**
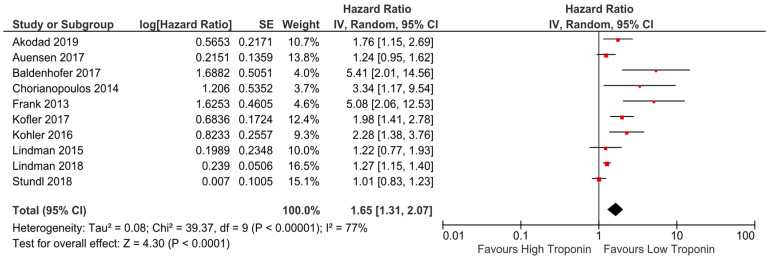
Forest plot of hazard and risk ratios of all-cause mortality for high vs. low levels of baseline Troponin using a random-effect model. This indicates that when comparing groups of high vs. low Troponin there was 1.65 times the risk of mortality in the higher group. CI: confidence interval; IV: inverse variance.

Of the studies analysing Troponin in the systematic review, ten reporting an effect size for high vs. low levels baseline Troponin were included in a meta-analysis. Pooled analyses demonstrated a statistically significant increase in all-cause mortality for high vs. low levels of baseline Troponin (pooled HR 1.65; 95% CI 1.31 to 2.07; *p* < 0.0001; Figure 2).

Most of the studies included in the meta-analysis were of at least fair quality (Supplementary Material, Table S3). I^2^ greater than 50% suggests substantial heterogeneity. Sensitivity analysis by excluding studies in turn did not significantly alter the results. The funnel plot generated showed some asymmetry (Supplementary Material, Figure S4).

### 3.4. Galectin-3

Galectin-3 belongs to the ß-galactoside binding protein family. It is expressed in leukocytes and fibroblasts and is released in response to myocardial inflammation and fibrosis [10]. Overall, five studies were systematically reviewed which analysed Galectin-3, including 1007 participants. Of these studies, three were large and all were observational with a follow-up over one year. The average age of participants was 79 years and 57% were male (Supplementary Material, Table S5). All studies reported all-cause mortality excluding one which exclusively reported MACE [59]. Galectin-3 was found to be significantly associated with all-cause mortality in all that reported it, with an average two-fold increased risk of death in those with higher baseline levels of Galectin-3 [43,66,75,76]. This significance only remained after multivariate adjustment in one study [75], with the significance abolished after adjustment for age [66], eGFR [76] and STS score [43] in the other studies. In the one study that reported MACE, Galectin-3 was found to be significantly associated with MACE; however, this did not remain on adjustment for confounding factors [59].

**Figure 3:**
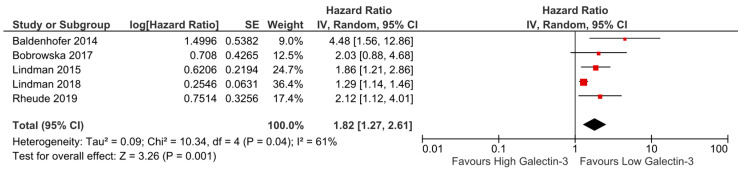
Forest plot of hazard and risk ratios of all-cause mortality for high vs. low levels of baseline Galectin-3 using a random-effect model. This indicates that there was 1.82 times the risk of mortality associated with the high-level group. CI: confidence interval; IV: inverse variance.

All five studies analysing Galectin-3 and all-cause mortality reported an effect size for high vs. low levels baseline Galectin-3, therefore were included in a meta-analysis. Pooled analysis demonstrated a statistically significant increase in all-cause mortality for high vs. low levels of baseline Galectin-3 (pooled HR 1.82; 95% CI 1.27 to 2.61; *p* = 0.001; Figure 3). All the studies included in the meta-analysis were of at least fair quality (Supplementary Material, Table S3); however, I^2^ was greater than 50% suggesting substantial heterogeneity. Sensitivity analysis by excluding studies in turn did not significantly alter the results. The funnel plot generated appeared slightly asymmetrical (Supplementary Material, Figure S5).

### 3.5. Other Biomarkers

The remaining 37 studies included in the systematic review looked at a range of less prominent biomarkers and are included in Supplementary Material, Table S6.

## 4. Discussion

Whilst individual studies have reported on multiple biomarkers [77,78], this is the first systematic review and meta-analysis to date that assesses the association of multiple biomarkers with mortality in a wider population of patients with AS.

The present analysis identified that high levels of baseline BNP, NT-proBNP, Troponin and Galectin-3 are associated with an increased risk of all-cause mortality in patients with AS. The strongest predictor of all-cause mortality was BNP with a more than double increased risk of death in participants with elevated baseline levels. This was not unexpected as BNP is released in response to ventricular stretch, which ultimately leads to myocardial injury and fibrosis.

NT-proBNP, Troponin and Galectin-3 were also found to be important determinants of mortality, where participants with elevated baseline levels had just under double the risk of death compared to those with lower levels at baseline. Troponin has also frequently been shown to predict mortality and, although the data is less robust compared to natriuretic peptides, many studies found baseline Troponin to be predictive of worse outcomes than BNP or NT-proBNP [15]. This may be because it is closely related to the degree of myocardial fibrosis [60], but this was not reflected in our analysis. Similarly, Galectin-3 participates in myocardial inflammation and fibrosis and was found to predict mortality as well as increase the predictive ability of NT-proBNP in one study [75]. Previous data have demonstrated a close link between Galectin-3 and accelerated cardiac hypertrophy in the pressure-overloaded myocardium, resulting in adverse myocardial remodelling and dysfunction [79]. Additionally, Galectin-3 has been associated with increased fibroblast activity and extracellular matrix [80], a pathophysiological process that has a crucial role in the disease progress and potentially precedes symptom occurrence in patients with AS. Given the fact that myocardial hypertrophy and fibrosis herald the presentation of symptoms and are correlated with adverse events [3], these biomarkers potentially have a critical role in risk stratification and are of significant prognostic value.

Other biomarkers included in the systematic review seemed to have a prognostic effect, but these are generally only available in the research setting, therefore unlikely to be implemented for routine use in clinical practice. In this regard, further research is required to show whether they have an additive benefit over and above other biomarkers.

Identification of biomarkers that associate with poor prognosis in patients with AS is of paramount importance in clinical practice to enable careful identification of patients for intervention, where treatment benefits outweigh the risks. Biomarkers such as natriuretic peptides may be raised in the elderly without AS and in some cohorts BNP is not elevated in the presence of decompensation [81], which creates difficulties when using one biomarker as an indication for intervention. For this reason, it is very possible that use of multiple biomarkers in combination will have a greater predictive ability than a single biomarker alone. Baldenhofer et al. found that a combination of NT-proBNP, mid-regional pro-adrenomedullin and mid-regional pro-atrial natriuretic peptide was a stronger predictor of 1-year mortality (HR, 7.03; *p* = 0.001) than using NT-proBNP (HR, 4.94; *p* = 0.013), mid-regional pro-adrenomedullin (HR, 3.34; *p* = 0.037), and mid-regional pro-atrial natriuretic peptide alone (HR, 4.94; *p* = 0.013) [55], which should be taken into account when implementing into clinical practice.

Mounting evidence has demonstrated that chances of survival, symptom improvement and improved quality of life are worse if valve replacement is performed late [82,83], suggesting that many individuals with severe symptomatic AS should be referred for earlier intervention. The high morbidity and mortality risk is likely driven by irreversible maladaptive LV remodelling and myocardial fibrosis and timely identification of those who would benefit from earlier intervention than specified in the guidelines is therefore paramount.

Biomarkers provide a relatively cheap and easily accessible way of identifying those with markers of poor prognosis such those with a maladaptive response to pressure overload or early signs of fibrosis. By identifying a single or group of biomarkers that closely associates with decompensation and/or poor prognosis, patients could undergo serial monitoring in the community. This would undoubtedly reduce the number of patients that miss the “therapeutic window” for intervention and therefore introduce a cost-effective way to improve post-operative morbidity and mortality rates. Moreover, serial monitoring of biomarkers may be advantageous in the post-operative period where consistently elevated biomarker levels after intervention determines which patients may benefit from additional medical therapy such as anti-arrhythmics or heart failure therapy [84].

These results have shown that many biomarkers are significantly associated with all-cause mortality and poor prognosis in those with AS. With this knowledge it is clear there is a potential role for the implementation of biomarkers in clinical practice; however, current literature falls short in defining the best way to do this. There is currently no randomized controlled trial (RCT) where patients with AS are randomised to receive surgical intervention based on a biomarker level. To clarify the exact role of biomarker testing in guidelines for AS, large adequately powered prospective studies are needed to determine the optimal cut-offs of biomarkers to use, as well as which biomarkers provide incremental value over existing methods of risk stratification.

## 5. Limitations

The findings of this meta-analysis have certain limitations. Firstly, the funnel plots show some asymmetry, perhaps due to no negative studies identified. However, physiologically an inverse association between the biomarkers and mortality would be unlikely. Secondly, substantial clinical and methodological heterogeneity was identified that may have affected biomarker level as well as outcomes. Moreover, the length of follow-up for mortality outcomes and estimates of effect greatly differed. Another important limitation is that the optimal cut-off values for baseline biomarker cannot be defined as there was a wide variation between studies in terms of assays and cut-off values used. This was accounted for by using a random-effects model for all meta-analyses to provide a more conservative estimate of the effect and considered high vs. low biomarker values as identified by the authors [85], which is an acceptable way of overcoming the differences. Meta-analysis of individualised patient data would have enabled us to identify mortality predictors more accurately, but this was not feasible within this timeframe. Although adjusted effect estimates such as RR and HR were reported in some studies, much of the mortality data was unadjusted, therefore our results must be interpreted with caution as they are subject to potential measured and unmeasured confounding. It is possible that the magnitude of this effect is small as, in the studies which reported both unadjusted and adjusted mortality data, the difference in values was minimal. Finally, bias was introduced from using study-specific cut-offs for biomarker level, which favours a positive result. Due to this, it is uncertain whether a particular cut-off level for each biomarker actually carries the estimated risk that we have reported from analysis.

## 6. Conclusions

Our results show that high levels of baseline biomarkers BNP, NT-proBNP, Troponin and Galectin-3 all predicted increased all-cause mortality in a wide population of patients with aortic stenosis. These results clarify that these biomarkers may have an important role in risk stratification of AS patients regardless of symptom status and could be used to refine optimal timing of intervention with a potential added benefit of using multiple biomarkers in combination. Although currently used by certain clinicians to guide their practice, further research is required to implement biomarkers into routine clinical practice to prevent the irreversible and severe consequences that result from undergoing late surgical intervention.

## Data Availability

All data are available from the corresponding author on request.

## Supplementary Materials

The following are available online at https://www.mdpi.com/article/10.3390/medsci9020029/s1, Figure S1: Search strategy, Figure S2: PRISMA 2009 Flow Diagram, Figure S3: Funnel plots for BNP and NT-proBNP meta-analyses, Figure S4: Funnel plot for Troponin meta-analysis, Figure S5: Funnel plot of Galectin-3 meta-analysis, Table S1: Characteristics of included studies reporting BNP, Table S2: Characteristics of included studies reporting NT-proBNP, Table S3: Newcastle-Ottawa Scale quality assessment for included studies, Table S4: Characteristics of included studies reporting Troponin, Table S5: Characteristics of including studies reporting Galectin-3, Table S6: Characteristics of included studies reporting other biomarkers.
