# Supplementary material for: Biomarkers Associated with Mortality in Aortic Stenosis: A Systematic Review and Meta-Analysis
Source: Med Sci (Basel). 2021 May 17;9(2):29. doi: 10.3390/medsci9020029 (PMC8163007; doi:10.3390/medsci9020029)
Supplement: Supplementary file 1 [file medsci-09-00029-s001.zip › medsci-1198681-supplementary.pdf]

Supplementary material

Figure 1. Search strategy

Figure 2. PRISMA 2009 Flow Diagram

Figure 3. Funnel plots for BNP and NT-proBNP meta-analyses

Figure 4. Funnel plot for Troponin meta-analysis

Figure 5. Funnel plot of Galectin-3 meta-analysis

Table 1. Characteristics of included studies reporting BNP

Table 2. Characteristics of included studies reporting NT-proBNP

Table 3. Newcastle-Ottawa Scale quality assessment for included studies

Table 4. Characteristics of included studies reporting Troponin

Table 5. Characteristics of including studies reporting Galectin-3

Table 6. Characteristics of included studies reporting other biomarkers

Figure 1. Search strategy

[(aortic stenosis) AND (blood biomarker OR biomarker OR BNP or troponin OR trop OR ST2 OR Galectin OR Osteopontin OR osteoprotegerin)].

Figure 2. PRISMA 2009 Flow Diagram

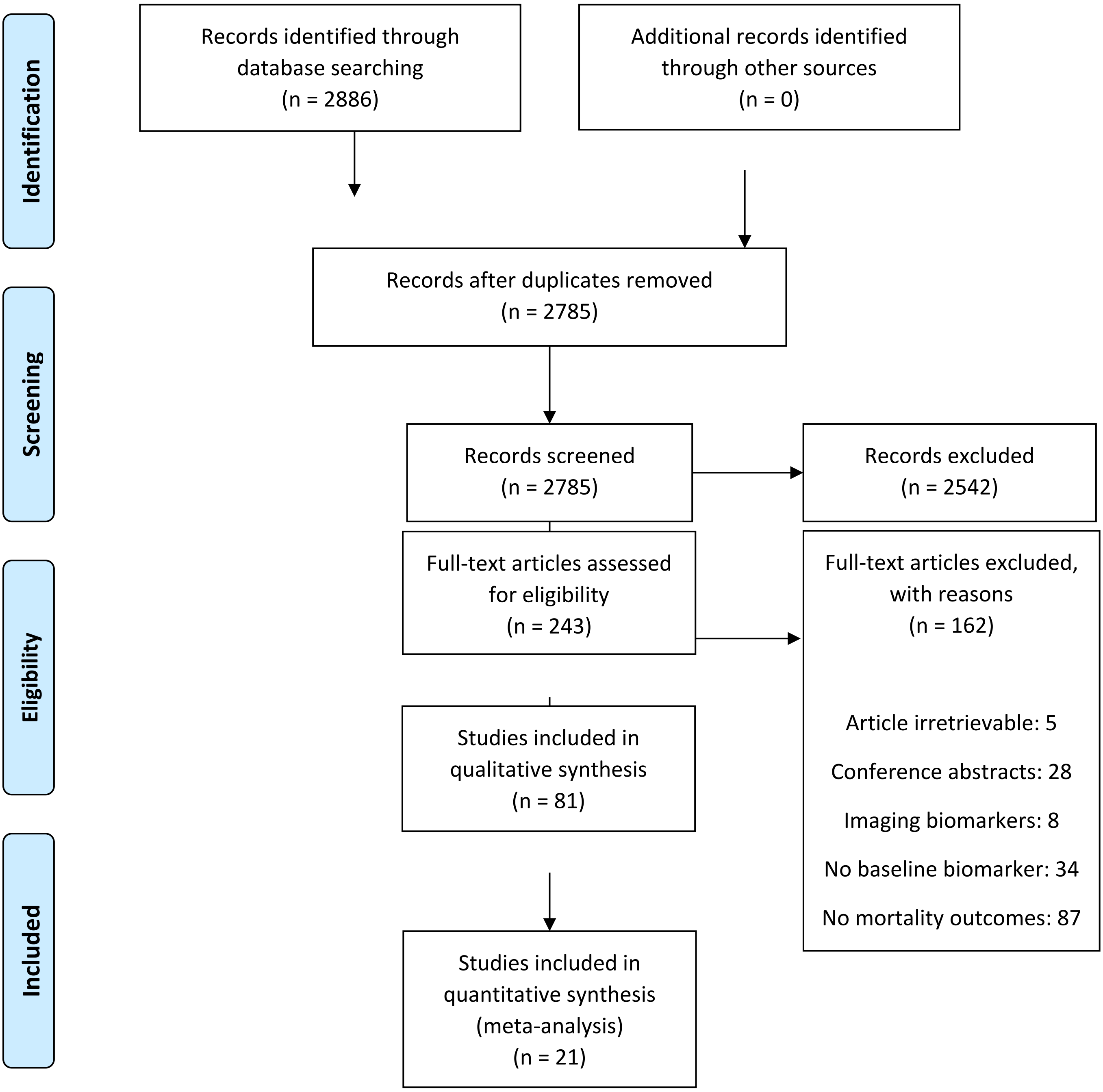

Figure 3. Funnel plots for BNP (A) and NT-proBNP (B) meta-analyses

(A) BNP studies

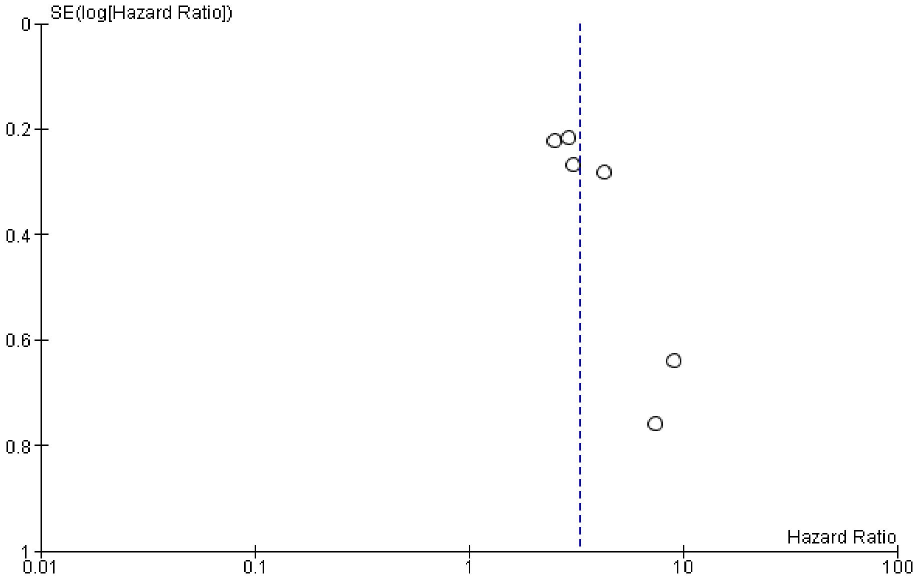

(B) NT-proBNP studies

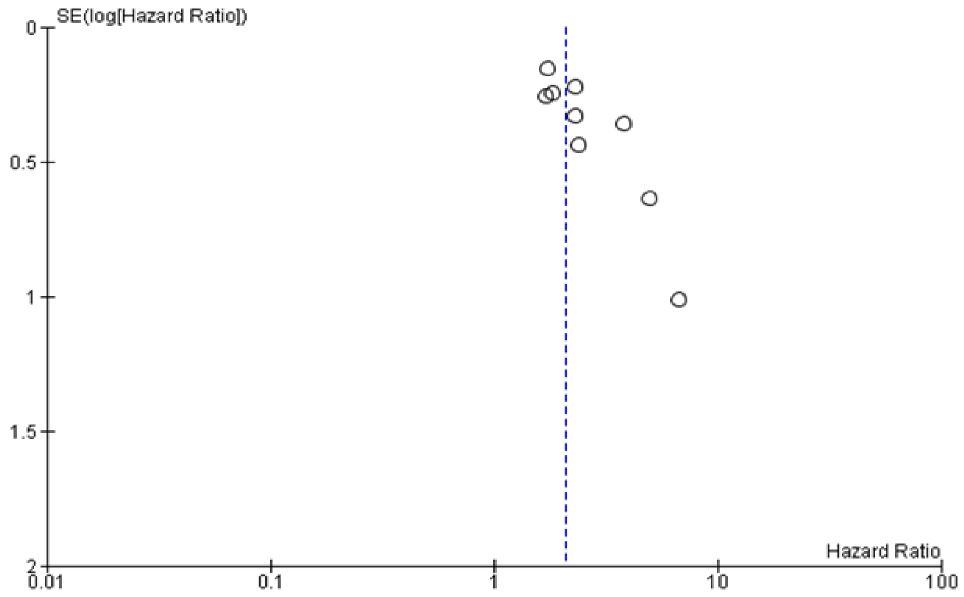

Figure 4. Funnel plot for Troponin meta-analysis

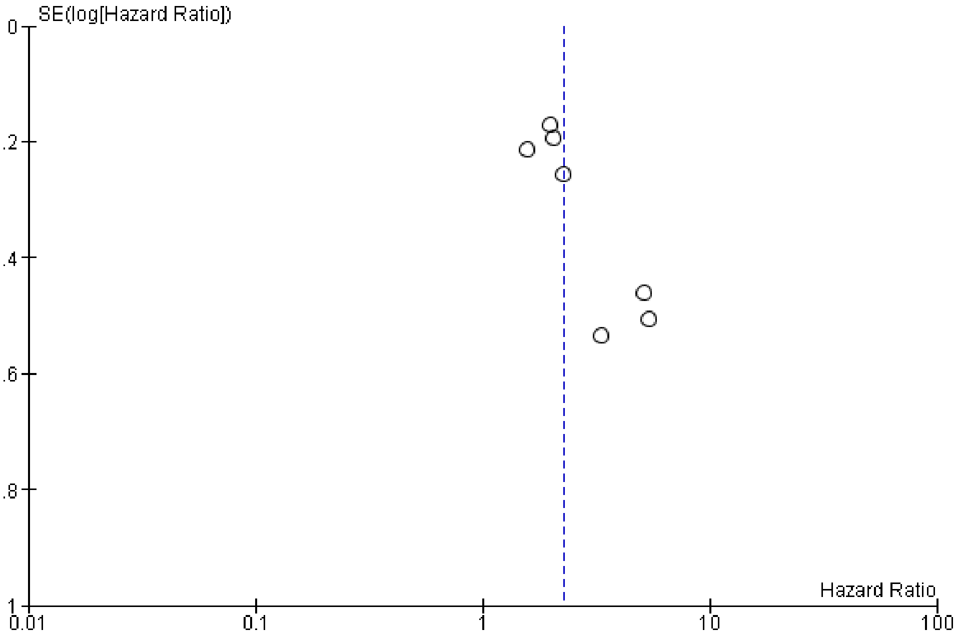

Figure 5. Funnel plot of Galectin-3 meta-analysis

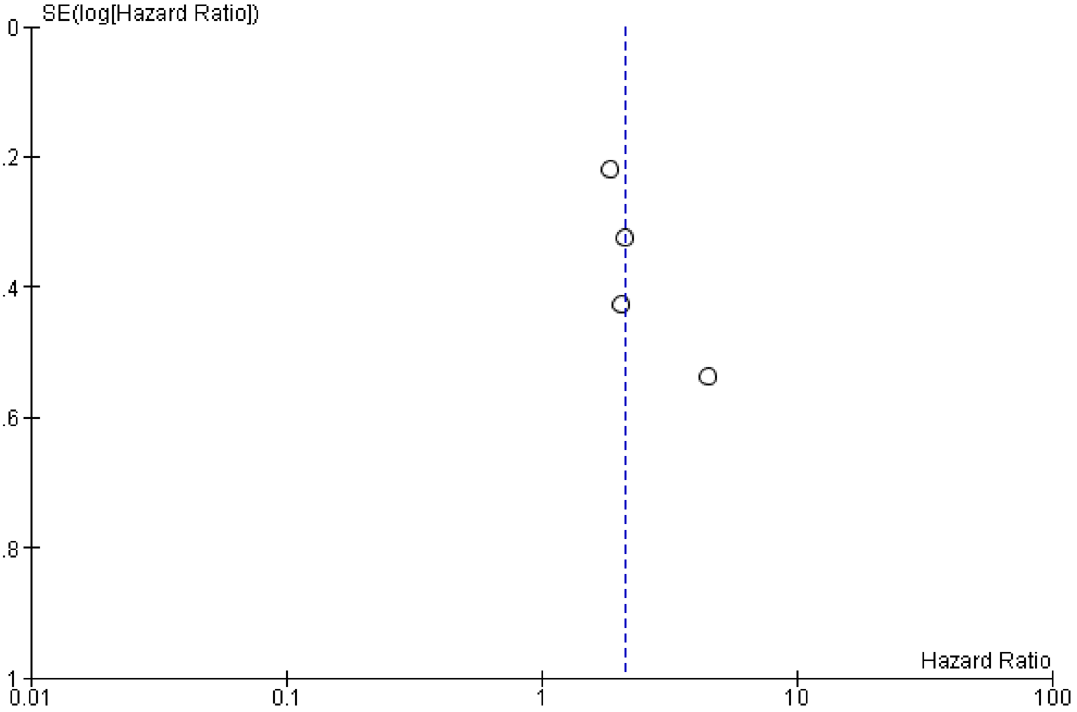

Table 1. Characteristics of included studies reporting BNP

Author/Year	*N*	Population	Follow-up	Age (years)	Male (%)	AS severity (N)	Main findings
Abramowitz Y et al. 2015[16]]	780	Severe AS undergoing TAVI	13.1 +/- 8.6 months (mean)	Low BNP: 82 (77-86) Mid and high BNP: 84 (78-89)	465 (59.6%)	Severe: 780	Univariate analysis was not significant for Mid vs. low BNP tertiles (HR 1.77 (0.96-3.26), p = 0.07) but was significant for High vs. low BNP tertiles (HR 4.29 (2.47-7.44), p<0.001) and BNP pg/ml (HR 1.05 (1.04-1.07), p <0.001). After multivariate analysis, Mid vs. low BNP tertiles was not significant (HR 1.58 (0.80-3.12), p = 0.19) but High vs. low BNP tertiles was significant (HR 3.26 (1.64-6.48), p = 0.001).
Andrinopoulou ER et al. 2015[17]	191	Severe AS	2 years	72.6 (SD 11.4)	118 (62%)	Severe: 191	From the joint model with the outcome death, they observed that higher BNP (Coef 0.5, Exp(coef):HR 1.65, Se(coef) 0.3, p=0.0962) at a specific time point tended to be associated with death. They concluded BNP had a good predictive capability for death, and little added value for the prediction of AVR.
Antonini-Canterin F et al. 2008[37]	64	Patients with AS (valve area 0.9 ± 0.3 cm2)	8 months (4.5–10 months)	76 ± 9	45.3%	N/A	Composite end-point was a composite of CV death, urgent AVR, hospitalization for congestive HF. BNP levels were significantly higher at baseline in the 18 patients who reached one of the pre-specified end-points during follow-up: 981 ± 821 vs. 142 ± 158 pg/mL for BNP (p=0.001). Patients with BNP values ≥254 pg/mL had an event-free survival at 12 months of 93% and 35% respectively (p<0.001).
Ben-Dor I et al. 2013[18]	289	High-risk patients with severe AS referred for TAVI	319 days (110-655) (median)	Low BNP: 82.9 ± 6.4 Mid BNP: 83.5 ± 6.6 High BNP: 81.0 ± 11.3	127 (43.9%)	Severe: 289	BNP was split into tertiles: Lowest: 273 ± 122pg/mL, Middle: 878 ± 272, Highest: 2761 ± 1127. On univariate analysis, log BNP was associated with mortality (HR 1.5, 95% CI 1.25-1.74, p<0.001), which didn't remain after multivariable adjustment. Patients who died had a median BNP level of 1,100 pg/dl (range 497 to 2,200) compared with 588 pg/dl (range 320 to 1,480) in survivors.
Bergler-Klein J et al. 2004[19]	130	Severe AS	377 ± 150 days	70 ± 12	67 (51.5%)	Severe: 130	By univariate analysis, preoperative BNP (P<0.05) was a significant predictors of survival, which didn't remain on multivariate analysis. Preoperative BNP (P<0.05) was a significant predictor of postoperative symptomatic status (NYHA class <II versus >II) by univariate analysis, which didn't remain so after multivariate analysis.
Bergler-Klein J et al. 2007[20]	69	Low-flow, low-gradient AS	411 ± 343 (1 to 1147 days)	70 ± 10	56 (81%)	N/A	BNP ≥550 pg/mL (P<0.001) was a significant predictors of outcome for the total cohort. Cumulative 1-year survival was 39±13% in medically treated patients with BNP ≥550 pg/mL but 100% in those with BNP <550 pg/mL. 1-year survival was lower in patients with BNP ≥550 pg/mL (50±14%) compared with those with BNP <550 pg/mL (100%).
Capoulade R et al. 2014[38]	211	Asymptomatic AS with normal LVEF	1.8 ± 1.3 years (mean)	68 ± 11	64%	Moderate: 54 (26%) Severe: 157 (74%)	The study end-point was the occurrence of death or AVR motivated by development of symptoms or LV dysfunction (ie, class I indication). Both resting and peak-ex BNP levels were associated with a 1.5-fold increase in risk of events (for each 100 pg/mL increase) (p<0.0001), which remained significant after adjustment.
Clavel MA et al. 2014[21]	1953	Patients with at least moderate AS	3.8 ± 2.4 years (mean)	76 ± 12	1081 (55%)	N/A	In univariable analysis, ln BNP and ln BNP ratio were associated with increased overall mortality (HR: 1.68 [95% CI: 1.60 to 1.77; p < 0.0001] and HR: 1.54 [95% CI: 1.47 to 1.62; p < 0.0001], respectively). After adjustment, ln BNP (HR: 1.40; 95% CI: 1.31 to 1.50; p < 0.0001) or ln BNP ratio (HR: 1.40; 95% CI: 1.31 to 1.50; p < 0.0001) was an independent predictor of mortality after diagnosis.

Dahou A et al. 2018[22]	98	Low-flow low-gradient AS (TOPAS study)	2.8 years (median)	73.7 ± 9.8	74 (75%)	N/A	2-year mortality was higher in group C (41 ± 9%) than in group B (23 ± 7%) and group A (5 ± 4%) (p = 0.002). Having either BNP or Hs-TnT raised and having both BNP and hs-TnT raised were significantly associated with increased mortality (Group B: HR 5.0, 95% CI 1.68–21.39, p=0.0024) and (Group C: HR 6.85, 95% CI 2.37–28.98, p=0.0001), respectively. This was similar after adjustment for the presence of renal failure (HR: 5.13; 95% CI = 1.37 to 25.30; p = 0.01).
Goodman A et al. 2016[23]	531	Patients with AS with aortic valve area <1.3 cm2	4.7 +/- 2 years	71 (12)	58%	Moderate: 72 (16%) Severe: 459 (86%)	Multivariable cox proportional hazard analysis for all-cause mortality showed BNP (for every 10 pg/mL increase) was associated with mortality (HR 1.16 (1.09–1.23), p<0.001 (stepwise fashion)) which remained when STS score entered in model (HR 1.14 (1.08–1.22), p<0.001). Multivariable Cox Proportional Hazard Survival analysis, for the secondary outcome (CV mortality and death due to unknown causes) showed that every 10 pg/mL increase in BNP (1.08 [1.06– 1.11]) was an independent predictor for secondary outcome.
Gotzmann M et al. 2014[24]	226	Symptomatic severe AS at high surgical risk	28 ± 549 days	80 ± 6	94 (42%)	Severe: 226	On univariate analysis, plasma BNP (>475 pg/ml) was associated with mortality (HR 1.001, 95% CI 1.000-1.002, p<0.001). This remained significant on multivariate analysis (HR 3.049, 95% CI 1.804-5.151, p<0.001). When using the cut-off point of BNP >328 pg/ml, sensitivity was 83% and specificity 67%. A plasma BNP level >328 pg/ml after 30 days was associated with all-cause mortality (HR, 8.125; 95% CI 3.097-21.318; p <0.001).
Henri C et al. 2016[39]	69	Moderate AS and preserved LVEF	30 ± 19 months	70 ± 12	42 (61%)	N/A	The primary endpoint was the occurrence of the first composite endpoint defined as the occurrence of symptoms, AVR or CV death. Patients with higher annualized BNP changes (> 20 pg/mL/y) had a significantly lower cardiac event-free survival (P < 0.001). Using the multivariate Cox proportional hazards model, higher annualized BNP changes were significantly associated with increased risk of cardiac events (HR 2.73, 95% CI 1.27-5.86; p=0.010) after adjustment for age, dyslipidemia, and echocardiographic parameters.
Iwahashi N et al. 2011[40]	109	Severe AS	44 +/- 10 months	68.1 +/- 10.6	53 (49%)	Severe: 109	Composite endpoint was CV death and readmission due to HF and stroke. An event-free survival rate was significantly higher in patients with BNP ≤312 pg/mL than BNP >312 pg/mL (P = 0.001). Univariate and multiple logistic regression analyses for the prediction of perioperative complication for BNP >312 pg/mL: univariate OR 7.79, (95% CI 2.69–26.44), p<0.0001, multivariate OR 5.58, (95% CI 1.82–20.16), p= 0.002.
Kefer J et al. 2010[25]	58	Severe AS undergoing TAVI	30 days	84 ± 5 years	36 (62%)	Severe: 58	In univariate analysis both change in BNP at 24 hours and baseline BNP were associated with mortality (HR 1.005, 95% CI (1.002–1.008), p=0.001) and (HR 1.001, 95% CI (1.000–1.002), p=0.002), respectively. This association remained significant for both after multivariate analysis (HR 1.001, 95% CI (1.000–1.0002), p=0.01) and (HR 1.006, 95% CI (1.002–1.0009), p=0.001).
Kim JB et al. 2017[26]	112	Severe AS undergoing TAVI	679 days (median)	84.0 ± 8.5	66 (59%)	Severe: 112	Although not found to be independently correlated with mortality in multivariate analysis, BNP showed significant difference in survival based on predefined cut-off values of 250 pg/mL and 6 ng/L, respectively. Despite strong correlation of BNP with LV functional recovery after TAVR on univariate analysis, this didn't remain significant after multivariate analysis.
Koskinas KC et al. 2015[27]	340	Severe native-vale AS undergoing TAVI	2 years	83.2 ± 4.8	145 (43%)	Severe: 340	On univariate analysis, BNP was associated with mortality (HR 2.50, 95% CI 1.62 to 3.86, p<0.001). After multivariate adjustments, high baseline BNP was associated with a higher risk of all-cause death (adjusted HR 3.16, 95% CI 1.84-5.42, p<0.001). High baseline BNP was associated with a high risk of CV- death at 2 years (adjusted HR 3.37, 95% CI 1.78-6.39, p<0.001).
Lancellotti P et al. 2010[41]	126	Asymptomatic patients with severe AS	20.3 +/- 18.7 months	No events: 68 +/- 10 Events: 67 +/- 12	75 (59.5%)	Severe: 126	The combined end point included onset of symptoms, CV death, and AVR. On multivariate Cox regression analysis, BNP was associated with the predefined outcome (p=0.012). Using receiver operating characteristics curve analysis, BNP of >61 pg/ml was identified as the best cut-off value to predict events. BNP was associated with the end-point in univariate p<0.0001 and multivariate (HR 1.001, 95% CI 1-1.003, p=0.012) analysis.

Lim P et al. 2004[28]	70	Severe AS	308 days (11–472)	74 years [62–82]	40 (57%)	Severe: 70	Univariate analysis showed that log BNP was significantly associated to poor outcome. By multivariable analysis (including log BNP, age, and NYHA class III), only BNP levels remained significantly associated to mortality (Log BNP HR 3.0, 95% CI 1.2-7.7, p=0.02). The multivariable logistic regression revealed that only BNP values were predictive of symptomatic AS (OR=3.4 [1.6–7.3], p<0.01). Optimal cut-off value of BNP to detect the presence of symptoms was 66 pg/ml with a sensitivity, specificity and accuracy of 84%, 82% and 84%, respectively.
Nakatsuma K et al. 2019[29]	387	Severe asymptomatic AS not referred for AVR	1190 (IQR: 732–1540) days (median)	Group 1: 75.6 ± 8.9<br>Group 2: 80.0 ± 8.4<br>Group 3: 83.6 ± 8.1<br>Group 4: 83.7 ± 8.3	159 (41.1%)	Severe: 387	Participants were split into BNP level: Group 1: BNP<100 pg/mL, Group 2: 100≤BNP<200 pg/mL, Group 3: 200≤BNP<300 pg/mL and Group 4: BNP>300 pg/mL. On univariate analysis, increasing BNP level was associated with all-cause mortality: Group 4 (HR: 4.37, 95% CI 2.62-7.16, p<0.001), Group 2 (HR: 2.00, 95% CI: 1.24-3.19, p=0.005). Primary outcome measure was a composite of AV-related death and HF hospitalisation. Univariate analysis found that increasing BNP level was associated with the composite endpoint: Group 2: HR 2.30, 95% CI 1.26-4.14, p=0.007 and Group 4: HR 5.86, 95% CI 3.13-10.76, p<0.001. After confounder adjustment, the risk of primary outcome measure was incrementally greater with increasing BNP levels (HR: 1.97, 95% CI: 0.97 to 3.98, p=0.06; HR: 3.59, 95% CI: 1.55 to 8.32, p=0.03 and HR: 7.38, 95% CI: 3.21 to 16.9, p<0.001, respectively.
Nessmith MG et al. 2005[30]	124	AS patients undergoing clinical evaluation	292 +/- 276 days	78 +/- 10	62 (50%)	N/A	When patients were stratified with BNP values of 296 and 819 pg/ml, 1-year mortality rates for the lower, middle, and upper tertiles were 6%, 34%, and 60%, respectively (RR per 1 tertile increment 2.9, 95% CI 1.9 to 4.6, p<0.001). After adjustment for symptoms, the predictive value of BNP continued to provide significant increased risk for death (RR per 1 tertile increment 2.4, 95% CI 1.5 to 4.1, p<0.001). After adjustment for univariate predictors, the predictive value of BNP for death remained significant (RR per 1 tertile increment 2.8, 95% CI 1.5 to 5.3, p<0.01). A stepwise Cox regression model including all clinical variables revealed that death was predicted by BNP (RR per 1 tertile increment 3.5, 95% CI 2.1 to 6.0, p<0.001).
O'Sullivan CJ et al. 2015[31]	340	Severe native valve AS who underwent TAVI	30 days	83.2 ± 4.8	145 (43%)	Severe: 340	As compared with the low BNP tertile group, high BNP tertile patients had a significantly higher incidence of all-cause (HR 7.41, 95% CI: 1.68-32.60, p=0.001) mortality at 30 days. As compared with the low BNP tertile group, high BNP tertile patients had a significantly higher incidence of cardiac (HR 5.82, 95% CI: 1.29-26.27, p=0.006) mortality at 30 days. Primary endpoint MACE, a composite of all-cause mortality, major stroke and myocardial infarction at 30 days. There was a significantly higher incidence of MACE among high BNP tertile patients as compared to low BNP tertile patients (HR 9.04, 95% CI: 2.09-39.14, p<0.001).
Parenica J et al. 2012[32]	42	High-risk patients undergoing AVR	1 year	82 (75; 89)	13 (31%)	Severe: 42	ROC analysis for prediction of 1-year mortality in those who underwent SAVR and TAVI for BNP was Sig. 0.376, AUC 0.614, 95% CI 0.343;0.885. A combined endpoint was defined as the occurrence of any clinical endpoint (either safety or efficacy endpoint) at 0–365 days. The combined endpoint analysis reflected the amount of patients experiencing at least one clinical endpoint.
Pedrazzini GB et al. 2008[33]	144	Symptomatic AS referred for AVR	17.7 months	79 +/- 9	84 (58%)	Severe: 144	Patients with logES >10.1% (upper tertile) had a higher risk of dying over time (HR 2.86, p 0.037), as had patients with BNP >312 pg/ml (HR 9.01, 95% CI 2.57-31.61, p <0.001). In the univariable Cox analysis, BNP (log scale) and BNP >312 pg/mL were associated with all-cause mortality (HR 2.31, 95% CI 1.45–3.69, p<0.001, C statistic 0.80, AIC 138.8) and (HR 9.01, 95% CI 2.57–31.61, p<0.001, C statistic 0.75, AIC 137.7), respectively. At the bivariable analysis, BNP >312 pg/mL and BNP (logscale) predicted death (HR 8.21, 95% CI 2.20-30.64, p=0.002) and (HR 2.02, 95% CI 1.22-3.36, p=0.006), respectively.
Ramchand J et al. 2019[34]	127	Patients with AS	5 years (IQR: 0.19 to 5 years)	75 +/- 11	81 (64%)	Mild: 25%<br>Mod: 24%<br>Severe: 51%	Patients with BNP activation had a significantly increased likelihood of death (HR: 4.64; 95% CI: 2.06 to 10.42; p < 0.001). All-cause mortality was highest in those with the combination of elevated plasma ACE2 activity and BNP clinical activation (HR: 13.78; 95% CI: 3.97 to 47.8; p < 0.001).

Sato K et al. 2019[35]	193	Severe symptomatic AS referred for TAVI	1,331 days (IQR: 632 to 1,678) (median)	80 +/- 11	114 (59.1%)	Severe: 193	In a univariable model, BNP as a time-dependent covariate was a significant predictor of survival (HR 1.24, 95%CI: 1.04-1.47, p=0.017). In a multivariable model, BNP as a time-dependent covariate remained significant after adjusting for STS score (HR 1.20, 95%CI: 1.01-1.44, p =0.041), and baseline BNP levels (HR 1.33, 95%CI: 1.01-1.73, p = 0.040), or both (HR 1.32, 95%CI: 1.001-0.73, p=0.049). Kaplan-Meier curves show that patients with high BNP (> 400 pg/ml) at 1-year post TAVR had poorer survival after TAVR (Log-rank p = 0.03).
Wernly B et al. 2017[36]	274	Symptomatic severe AS	1 year	80 ± 0.5 years	125	Severe: 274	In a Cox regression analysis, BNP (changes per pg/mL) was not associated with increased mortality (HR 1.0001, 95% CI 0.9999-1.0003, p=0.28).

Table 2. Characteristics of included studies reporting NT-proBNP

Author/Year	*N*	Population	Follow-up	Age (years)	Male (%)	AS severity (N)	Main findings
Agoston-Coldea L et al. 2019[42]	52	Severe AS undergoing AVR	386 (60-730) days	66 (7.5)	29 (55.7%)	Severe: 52	In univariate analysis, NT-proBNP was not associated with mortality (unadjusted HR 1.00 (0.99–1.01), p value NS). Outcomes were defined as the composite of MACEs. In Cox regression analysis, only reduced LAS (HR 1.33, 95% CI (1.01 to 1.74), p < 0.01) and the presence of LGE (HR 11.3, 95% CI (1.82 to 70.0), p < 0.01) were independent predictors for MACEs. A stepwise multivariate Cox regression model was constructed, including age, 6MWD, E/E'ratio, LVEF, LAS and the presence of LGE. Only reduced LAS (HR 1.33 (95% CI 1.01–1.74; chi-square: 15.1, p < 0.001) and LGE (HR 11.3 (95% CI 1.82–70.2); chi-square: 24.3, p < 0.001) were independent predictors for the combined end-point.
Agoston-Coldea L et al. 2018[62]	42	Severe AS	347 days (range 60-450 days)	73 (6)	23 (54.8%)	Severe: 42	Composite of MACEs included sudden cardiac death, non-fatal myocardial infarction, sustained ventricular arrhythmias, atrial arrhythmias, and hospitalization for heart failure. In univariate analysis, NT-proBNP was associated with MACE (unadjusted OR 1.02 (1.01-1.06), p=0.004). This remained significant in multivariate analyis (adjusted OR 1.18(1.07-1.42), p<0.01). Kaplan–Meier analysis showed that freedom from MACEs was significant in patients who exhibited high levels NT-proBNP [HR 5.22 (95% CI 1.85-14.51), p=0.001].
Anantha-Narayanan M et al. 2019[43]	222	Veterans undergoing TAVI	2.3 years	78 ± 8 years	220 (99%)	N/A	Long-term mortality was 5.26% for patients with no or Grade I DD, 25% for patients with indeterminate DD and 28% in patients with DD Grades II–III. NT-pro BNP <50th percentile had lower long-term mortality compared to NT-pro BNP > 50th percentile (9.8% vs. 26.4% in p = 0.05). Advanced and indeterminate DD had increased long-term mortality (25–28% vs. 5%, p = 0.02).
Auensen A et al. 2017[44]	442	Severe AS referred for AVR evaulation	3 years	74 ± 11	249 (56%)	Severe: 442	Adjusted analysis showed that none of the studied biomarkers (NT–proBNP, hsTnT or hs–CRP) or any other covariates were associated with 3–year all–cause mortality following SAVR. Number of cardiac deaths were 47 of 79 (59.5%) at 3 years from inclusion, whereas 16 of 35 (45.7%) of operated patients and 31 of 44 (70.5%) of un-operated patients died cardiac deaths. Defined as all-cause death, TIA, stroke and MI. NT-proBNP was not found to be associated with MACE after TAVI.
Baldenhofer G et al. 2017[45]	100	Severe AS undergoing TAVI	1 year	78 ± 8	45 (45%)	Severe: 100	The single predictor model found that NT-proBNP was associated with all-cause mortality (HR 4.94, 95% CI 1.41–17.33, p = 0.013). This association remained on adjustment for age (HR 4.71, 95% CI 1.34–16.58, p=0.016), gender (HR 4.75, 95% CI 1.35–16.76, p=0.015) and GFR (HR 5.14, 95% CI 1.45–18.28, p=0.011). Cardiovascular events defined as CV death, non-fatal MI, and stroke or TIA. On univariate analysis, NT-proBNP was associated with CV events (HR 3.26, 95% CI 1.29–8.28, p=0.013). This association remained significant after adjustment for age (HR 3.14, 95% CI 1.23–7.99, p=0.016), gender (HR 3.09, 95% CI 1.21–7.91, p=0.018) and GFR (HR 3.32, 95% CI 1.29-8.55, p=0.013).
Bergler-Klein J et al. 2004[19]	130	Severe AS	377 ± 150 days	70 ± 12	67 (52%)	Severe: 130	By univariate analysis, NtBNP (P<0.001) was a significant predictors of survival, whereas age, presence of coronary artery disease, and aortic valve area were not. By multivariate analysis, NtBNP (P<0.001) remained the only independent predictor of survival. Preoperative NtBNP (P<0.01) were significant predictors of postoperative symptomatic status (NYHA class <II versus >II) by univariate analysis, but NtBNP (P<0.05) remained the only independent predictor by multivariate analysis. By univariate analysis, NtBNP (P<0.01) was a significant predictor of normal postoperative EF (EF <50% versus >50%). Both preoperative EF (P<0.05) and NtBNP (P<0.05) remained significant predictors of normal postoperative EF by multivariate analysis.

Chin CW et al. 2014[63]	131	Asymptomatic moderate to severe AS (SALTIRE study)	10.6 years	67 ± 10	91 (70%)	N/A	Composite endpoint was occurrence of AVR and cardiovascular deaths. NT-proBNP was not associated with AVR or cardiovascular deaths in both unadjusted (HR 1.15 per two-fold increment in NT-proBNP concentration; 95% CI, 0.86–1.53, P = 0.34) and adjusted analyses for age and sex (HR 1.14, 95% CI 0.80-1.60, p=0.47) and additional adjusting for dyspnoea (HR 1.13, 95% CI 0.80-1.60, p=0.49).
Cho KI et al. 2016[64]	336	Severe calcific AS (newly diagnosed)	33 months	70.1 ± 12.0 years	166 (49.4%)	Severe: 336	MACE was defined as a composite of all-cause mortality, cardiac death and non-fatal myocardial infarction during the follow-up period. On univariate analysis, NT-proBNP was weakly associated with MACE (HR 1.00, (1.00-1.00), p<0.001).
Cimadevilla C et al. 2013[65]	361	Patients >70 years with at least mild AS	1.8 ± 0.8 years	79 ± 6 years	205 (57%)	Mild: 44 (12%) Moderate: 87 (24%) Severe: 230 (64%)	Event-free survival (composite endpoint of AS-related events). NT-proBNP values at baseline were lower in patients who remained asymptomatic than in patients who developed symptoms (319±431 vs 633±670 pg/ml, p=0.001). Survival curves showed that patients with baseline measurement of Nt-proBNP less than 300 pg/ml had a better outcome than patients with Nt-proBNP values between 300 and 700 pg/ml or above 700 pg/ml (91%, 77% and 59% event-free survival at 2 years, respectively, p=0.005).
Csordas A et al. 2015[46]	153	Severe AS undergoing TAVI	258 days (169–443)	82 (78–86)	76 (50%)	Severe: 153	Both proBNP, ng/L (per 1 unit increase) and proBNP (upper quartile) were found to be associated with all-cause mortality (HR 1.0 (1.0–1.1), p = 0.03) and (HR 3.1 (1.2–8.4), p=0.02), respectively. This association did not remain significant on bivariate analysis for MR-proADM and proBNP for both proBNP, ng/L (per 1 unit increase) (HR 0.9 (0.9–1.0), p<0.05) and proBNP (upper quartile) (HR 1.2 (0.3–4.0), p = 0.75). On multivariate analysis proBNP in the upper quartile was not associated with all-cause mortality (HR 1.2, 95% CI 0.3–4.0, p=0.75). Bivariate (MR-proADM + proBNP) (per 1 unit increase): HR 0.9, 95% CI 0.9–1.0, p=0.05.
Elhmidi Y et al. 2013[47]	373	Severe AS undergoing TAVI	12 ± 2 months	81 (77-85)	139 (37.2%)	Severe: 373	There were no significant differences in 30-day mortality (P=.84) and cardiac rehospitalization rate (P=.16) among the NT-proBNP tertiles but a higher 1-year mortality in the 3rd tertile (P=.024). In the univariable analysis, baseline (HR, 1.01; 95% CI, 1.001-1.02; P=.02) and post-treatment NT-proBNP (HR 1.02; 95% CI, 1.002-1.04; P=.04) were predictors for 1-year mortality. In the multivariable analysis, however, only baseline NT-proBNP and atrial fibrillation were identified as predictors for the 1-year mortality (HR, 1.02; 95% CI, 1.01-1.05; P=.006 and HR, 3.4; 95% CI, 1.25-9.5; P=.017, respectively).
Farre N et al. 2014[66]	237	Degenerative asymptomatic moderate to severe AS	18 (IQR 7-31) months	74 ± 9	120 (49%)	Moderate: 61 (26%) Severe: 176 (74%)	Event-free survival at 1 and 2 years was 87% and 79% in the first quartile, compared with 45% and 28% in the fourth quartile, respectively. The primary endpoint was the occurrence of a composite event of hospitalization for angina, syncope or heart failure, AVR surgery, or death from any cause. Cox regression for combined endpoint was NT-proBNP: HR 1.0, 95% CI 1.000-1.000, p=0.006
Ferrer-Sistach E et al. 2019[67]	58	High gradient ASAS	1 year ± 30 days	74.8 ± 8.4	33 (56.9%)	Severe: 58	Primary endpoint was a composite of CV death, new-onset symptoms, cardiac hospitalization, guideline-driven indication for valve replacement and CV death at 12m. On univariate analysis, primary endpoint was associated with NT-ProBNP (HR 2.24, 95% CI 1.48-3.39, p<0.001). In multivariable analysis, the primary endpoint only remained significantly independently associated with aortic regurgitation of ≥2 (P=0.01) and hs-TnT (P=0.007) (Table 2). ROC analysis revealed that the best cut-off value for hs-TnT was 10 ng/L.

Frank D et al. 2013[48]	107	Patients underoging TAVI	249 days (± 158 days)	81.63 ± 6.34	38.30%	N/A	Factors significantly associated with elevated hsTnT before TAVI included elevated NTproBNP (p = 0.001), high CRP (p b 0.001), as well as the clinical risk scores Euroscore, Euroscore II, and STS. On univariate analysis, NT-proBNP in the upper quartile (>4657.00 pg/mL)was borderline associated with all-cause mortality (HR 2.37; 95% CI [1.00, 5.64]; P=0.05).
Kaneko H et al. 2019[49]	717	Patients with severe AS and HF undergoing TAVI	Unknown	Responder: 80 ± 7 Non-responder: 81 ± 7	334 (47%)	Severe: 717	Kaplan–Meier curves showed that NT-proBNP nonresponders had lower survival rates after hospital discharge (logrank p<0.001). Multivariable Cox regression analysis revealed that NT-proBNP > 7500 pg/mL (HR 1.8; p=0.020; 95% CI 1.1–3.1) and NT-proBNP nonresponse (HR 2.3; p=0.001; 95% CI 1.4–3.9) were independently associated with higher mortality after hospital discharge
Kohler WM et al. 2016[50]	259	AS undergoing TAVI	290 (Q1=88; Q3=529) days	83 ± 6.1	44.80%	N/A	Higher STS score (p=0.014, cut-off 10%), Log ES category (p=0.002, cut-off 20%), higher NTproBNP levels (crude HR 1.82 (1.12-2.97) p=0.016) cut-off 5163,5 pg/mL), higher, and several VARC-2 criteria were all linked to increased TAVI mortality. The significance of NT-proBNP did not remain significant. Higher baseline hsTNT could be linked to kidney disease (p=0.028), pulmonary hypertension (p<0.001), higher overall risk scores (all p ≤0.001), increased NTproBNP levels (p=0.004), and several VARC-2 criteria (e.g., CV death; p=0.016).
Koskinas KC et al. 2015[27]	340	Severe native-vale AS undergoing TAVI	2 years	83.2 ± 4.8	145 (43%)	Severe: 340	After multivariate adjustments, high baseline BNP was associated with a higher risk of all-cause death (p <0.001) and cardiovascular death at 2 years (p <0.001). After multivariate adjustments, high baseline BNP was associated with a higher risk of all-cause death (adjusted HR 3.16, 95% CI 1.84-5.42, p<0.001). NT-pro-BNP outperformed all other single measurements as a predictor of all-cause mortality at 2 years. High baseline BNP was associated with a high risk of CV- death at 2 years (adjusted HR 3.37, 95% CI 1.78-6.39, p<0.001). Multivariable logistic regression analysis revealed that baseline NT-pro BNP≤ 7500 pg/mL (OR 3.2; p<0.001), amongst other things were independent determinants of NT-proBNP nonresponse after TAVI.
Krau NC et al. 2015[51]	217	Patients with severe AS undergoing TAVI	349 days (106–660)	81.8 ± 6.0 years	44.20%	Severe: 217	Analyses comparing the upper quartile with the lower three quartiles for biomarker levels revealed a HR of increased GDF15 for adverse outcome of 2.4 [95% confidence interval (CI) 1.5–3.9, P < 0.001] compared NT-proBNP: 1.71 (1.0–2.8) P = 0.037. The Wald test indicated that Log ES (P =0.001), Log ES II (P =0.018), STS score (P =0.019). NT-proBNP (P =0.037), and AFIB/flutter (P =0.02) demonstrated significance for predicting a negative outcome. In multiple Cox regression analysis, NT-proBNP did not remain significant when adjusted for GDF15.
Lindman BR et al. 2018[52]	665	Patients undergoing surgical AVR	10.7 years	71 (63–77)	621 (93%)	Severe: 665	The highest hazards for mortality were seen with higher levels of high-sensitivity cardiac troponin T, HE4, and GDF15. Standardized log2 transformed (unadjusted): hs-CTnT (HR: 1.34, 95% CI: 1.22-1.48, p<0.001), HE4 (HR: 1.86, 95% CI: 1.67-2.08, p<0.001), CA125 (HR: 1.27, 95% CI: 1.15-1.39, p<0.001), GDF15 (HR: 1.69, 95% CI: 1.52-1.88, p<0.001), NTproBNP (HR: 1.39, 95% CI: 1.24-1.56, p<0.001), hsCRP (HR: 1.28, 95% CI: 1.14-1.43, p<0.001), ST2 (HR: 1.30, 95% CI: 1.17-1.44, p<0.001).
Lindman BR et al. 2015[53]	345	Patients with severe AS	1.9 ± 1.2 years	78 (11)	194 (66%)	Severe: 345	Standardized log2 transformed NT-proBNP and NT-proBNP above median (>1402) were both significantly associated with mortality (HR 1.47, 95% CI 1.18-1.82, p<0.001) and (HR 2.29, 95% CI 1.47-3.58, p<0.001), respectively. This remained when Log2 Nt-proBNP was adjusted for STS (HR 1.21, 95% CI 0.95, 1.54, p=0.13) and clinical factors (HR 1.31, 95% CI 1.02, 1.67, p=0.032). This also remaines significant when NT-proBNP >1402 was adjusted for STS (HR 1.71, 95% CI 1.06, 2.74, p=0.027) and clinical factors (HR 1.77, 95% CI 1.11, 2.82, p=0.017).

López-Otero D et al. 2013[54]	85	Severe symptomatic AS undergoing TAVI	365 days (IQR: 189–660)	83 ± 5.43	54 (63.5%)	Severe: 65	Log pro-BNP showed a linear relationship with mortality, such that both pre-operative and long-term follow-up mortality rates were higher in patients with higher pro-BNP levels. On multivariate analysis, log pro-BNP was the only strong independent predictor of 30-day mortality [HR= 11 (95% CI: 1.51–81.3), p=0.018], while LES did not significantly correlate with mortality [HR=1.03 (95% CI: 0.95–1.10), p=0.483]. Univariate analysis for mortality at the end of follow-up for Log pro-BNP [HR 5.35 (95% CI 1.74–16.5), p=0.003]. Multivariate analysis shows that mortality during follow-up was associated with log pro-BNP [HR=4.51 (95% CI: 1.37–14.8), p=0.013].
Nguyen V et al. 2017[68]	809	At least mild degenerative AS	2 years	75 ± 10	620 (77%)	Mild: 80 (10%) Moderate: 180 (22%) Severe: 549 (68%)	In univariate analysis, Nt-proBNP was significantly associated with outcome (p = 0.01). However, Nt-proBNP was not predictive of outcome in multivariate analysis after adjustment for age, gender, rhythm and AS severity (p = 0.06, p = 0.20 and p = 0.15). On multiple regression model, independent determinants of Nt-proBNP overall were age (p = 0.0008), symptoms (p = 0.003), AVA (p = 0.005), rhythm (p = 0.007) and grade of diastolic function (p b 0.0001). Independent determinants of Nt-proBNP in patients with severe AS were age (p = 0.01), gender (p = 0.02), symptoms (p = 0.05), AVA (p = 0.001), history of CAD (p = 0.03) and diastolic function (p < 0.0001).
Parenica J et al. 2012[32]	42	High-risk patients allocated to TAVI or SAVR	1 year	82 (75; 89)	13 (31%)	Severe: 42	ROC analysis for prediction of 1-year mortality in those who underwent SAVR and TAVI was BNP: Sig. 0.376, AUC 0.614, 95% CI 0.343;0.885 and NT-proBNP: Sig. 0.590, AUC 0.569, 95% CI 0.327;0.812. Combined endpoint was occurrence of any clinical endpoint (either safety or efficacy endpoint) at 0–365 days. ROC analysis combined safety endpoint (0-365 days): Sig. 0.520, AUC 0.560, 95% CI 0.380-0.741.
Raposeiras-Roubín S et al. 2016[55]	54	Patients ≥ 90 years with severe AS	1.7 ± 1.5 years	N/A	N/A	Severe: 54	NT-proBNP was significantly associated with total mortality (HR 2.31 for the median of NT-proBNP, 95% CI 1.20-4.41; p=0.012), at the expense of cardiovascular mortality (HR 2.58 for the median of NT-proBNP, 95% CI 1.27-5.21; p=0.008; for non-cardiovascular death: HR 1.09 for the median of NT-proBNP, 95% CI 0.18-6.79; p=0.922). After multivariate analysis (table 1), NT-proBNP remained as a strong predictor of mortality at the expense of CV etiology, together with left ventricular systolic dysfunction.
Rheude T et al. 2019[69]	439	Severe AS undergoing TAVI	371 days [219-402]	81.0 (77.0-85.0)	240 (54.7%)	Severe: 439	Optimal cut-off to predict the primary endpoint was 2570 ng/L for NTproBNP (elevated). Primary endpoint was composite of all-cause mortality and readmission for worsening CHF during 1 year post-TAVI. Multivariate cox regression adjusted for baseline variable NT-proBNP >2570 ng/L: HR 2.12, 95% CI 1.12-4.38, p=0.022.
Rheude T et al. 2018[70]	363	Severe AS undergoing transfemoral TAVI	1 year	81 ± 6	197 (54%)	Severe: 363	Separately, all-cause mortality (14% [21 of 151] vs 3% (6 of 212]; p <0.001), cardiac death (10% [15 of 151] vs 2% [4 of 212]; p = 0.001), and readmission for CHF (20% [30 of 151] vs 5% [10 of 212]; p <0.001) were more frequent in case of elevated NTproBNP levels. Primary end point was all-cause death or readmission for worsening congestive heart failure within 1 year after TAVI. After multivariable adjustment, elevated NT-proBNP was independently associated with the primary end point (HR 2.12; 95% CI [1.12 to 4.38]; p = 0.022).
Røsjø H et al. 2014[56]	57	Moderate to severe AS	1287 days	75 ± 1	26 (46%)	N/A	On univariate analysis NT-proBNP was associated with all-cause mortality (HR 1.71, 95% CI 1.04–2.81, p=0.036). This remained significant on multivariate analysis (HR 2.20, 95% CI 1.17–4.14, p=0.014). High NT-proBNP and miR-210 levels, together with history of hypertension and increasing eccentric LV hypertrophy based on calculation of relative wall thickness, were associated with mortality in multivariate Cox proportional hazard regression analysis. The prognostic accuracy of miR-210 for all-cause mortality was comparable to the accuracy of NT-proBNP levels: AUC = 0.64 (95% CI 0.50–0.76) vs. AUC = 0.67 (0.53–0.79), respectively, p = 0.83.
Sinning JM et al. 2015[57]	310	Patients with AS undergoing TAVI	1 year	83.0 (77.0-86.0)	165 (53.4%)	N/A	Composite endpoint was a composite of 1-year death or rehospitalization due to cardiovascular disease (EuroSCORE II, AUC 0.690; GDF-15, AUC 0.682). Prediction of 1-year death for NT-proBNP was HR 1.4 (1.2-1.7), p<0.001. Also reported discrimination of outcome events: 1-year death: AUC 0.647, 95% CI 0.591-0.700, p<0.001 and 1-year death of hospitalisation: AUC 0.607, 95% CI 0.550-0.661, p=0.001.

Solberg OG et al. 2012[58]	136	Severe symptomatic AS	37 months (1 – 54 months)	74 ± 9	57%	Severe: 136	There was a strong association between NT-proBNP and all-cause mortality. The unadjusted HR when comparing quartile 4 with quartile 1 – 3 was 6.44 (2.17 – 19.09, p<0.001) for NT-proBNP. Multivariable analyses: [Log e] NT-proBNP: unadjusted HR 8.54 (4.01 – 18.17), p<0.001. On a combined model [Log e] HsTnT [Log e] NT-proBNP: unadjusted HR 1.96 (1.62 – 2.38), p<0.001, which persisted on multivariable analysis.
Stähli BE et al. 2015[59]	244	Severe symptomatic AS undergoing TAVI	277 [40–604] days	84 ± 7.1	122 (50%)	Severe: 244	In ROC analysis, NT-proBNP-ratio significantly predicted 1-year mortality (AUC: 0.72; CI: 0.64–0.80; P<0.001; sensitivity 81%, and specificity 56% at a cutoff level of 4.5) in the total patient cohort. Univariable Cox analysis, predictors of all-cause mortality in descending order of HR were NT-proBNPratio (above and below the median; HR 3.80, CI 1.88–7.74, P < 0.001), COPD (HR 2.43, CI 1.28–4.62, P = 0.007), and creatinine (HR 1.01, CI 1.00–1.01, P < 0.001). By multivariable Cox analysis, these three variables remained significant outcome predictors.
Stundl A et al. 2017[60]	461	Severe AS undergoing TAVI	1 year	81.3 ± 6.4	234 (50.8%)	Severe: 461	Analysis of baseline NT-proBNP levels revealed a nonsignificant difference in 30-day mortality (7.9% vs 4.7%, p = 0.189), and a statistically significant increase in 1-year mortality (39.5% vs 21.0%, p <0.001) for NT-proBNP levels >8145 pg/ml (Q4) vs NTproBNP levels ≤8145 pg/ml (Q1 to Q3). In univariate analysis, NT-proBNP was associated with mortality (OR 1.405, 95% CI 1.229-1.607, p<0.001), which did not remain significant on multivariate analysis.
Vale NC et al. 2018[61]	151	Symptomatic AS undergoing TAVI	1 year	81.5 ± 7.6	57 (38%)	N/A	Baseline and post-procedural NT-proBNP above 1350 and 2500 pg/ml, respectively, were both associated with one-year mortality. Multivariate Cox regression showed that only NT-proBNP higher than 2500 pg/ml (HR 5.9, 95% confidence interval 1.6-21.7, p=0.008) remained independently and negatively associated with one-year survival. Early variation in NT-proBNP levels (during the first month after TAVI) was highly heterogeneous and the absolute value did not differ significantly from baseline.
Weber M et al. 2006[71]	159	Patients undergoing evaluation for AS	902 (861–952) days	69 (12)	80 (50%)	Mild: 26<br>Moderate: 31<br>Severe: 102	Baseline NT-proBNP >640pg/ml predicted adverse outcome. Combined end point was CV death and rehospitalisation for acute HF. In Kaplan–Meier analysis baseline NT-proBNP values discriminated patients with an adverse outcome in the entire study group (HR 3.70, 95% CI 1.46 to 9.36, log rank test p = 0.006) and in the conservative group (HR 13.71, 95% CI 3.48 to 54.03, log rank test p , 0.001). In surgically treated patients baseline NT-proBNP didn't discriminate patients at high risk (HR 1.78, 95% CI 0.55 to 5.76, log rank test p = 0.335).

Table 3. Newcastle-Ottawa Scale quality assessment for included studies

Author	Selection	Comparability	Exposure/Outcome	Total	Quality of study
Abramowitz 2015[16]	***	**	**	7	Good
Akodad 2019[72]	***	*	**	6	Good
Baldenhofer 2014[78]	**	**	**	6	Fair
Baldenhofer 2017[45]	**	*	**	5	Fair
Bobrowska 2017[79]	***	**	**	6	Good
Chorianopoulos 2014[73]	***	*	***	7	Good
Frank 2013[48]	**	-	*	3	Poor
Gotzmann 2014[24]	**	*	**	5	Fair
Kofler 2017[74]	**	*	*	4	Poor
Kohler 2016[50]	**	*	***	6	Fair
Koskinas 2015[27]	***	*	***	7	Good
Krau 2015[51]	**	-	*	3	Poor
Lindman 2015[53]	***	*	**	6	Good
Nessmith 2005[30]	***	**	***	8	Good
O'Sullivan 2015[31]	***	-	**	5	Poor
Pedrazzini 2008[33]	***	*	**	6	Good
Raposerias-Roubin 2016[55]	**	*	*	4	Poor
Rheude 2019[69]	**	*	***	6	Fair
Stahli 2015[59]	**	**	*	5	Poor
Stundl 2017[60]	**	*	***	5	Fair
Vale 2018[61]	**	-	*	3	Poor

Table 4. Characteristics of included studies reporting Troponin

Author	*N*	Population	Follow-up	Age (years)	Male (%)	AS severity (N)	Main findings
Akodad M et al. 2019[72]	1390	Patients undergoing TAVI	360 days	83.4 ± 6.8	727 (52.3%)	N/A	In univariate and multivariate analysis, higher hs-TnT (T3 vs. T1) was significantly associated with all-cause mortality (HR 2.32, p<0.001) and (HR 1.76, 95% CI 1.15–2.68, p=0.009), respectively. HRs were adjusted (forward stepwise likelihood ratio) for procedure date and for baseline characteristics with a univariate P<0.10 for the outcome of 1-year mortality.
Auensen A et al. 2017[44]	442	Severe AS referred for AVR evaulation	3 years	74 ± 11	249 (56)	Severe: 442	Analysis using standardized log2 transformation showed that hs-TnT was not associated with 3–year all–cause mortality following SAVR (HR 1.24, 95% CI 0.95–1.62, p= 0.106). Adjusted analyses showed that hsTnT (HR, 1.51; 95% CI 1.11–2.05; P = 0.008) and left ventricular ejection fraction (HR 0.97; 95% CI 0.94–0.97, P = 0.043) was associated with MACE defined as all-cause death, TIA, stroke and MI for operated patients during 1 year.
Baldenhofer G et al. 2017[45]	100	Severe AS undergoing TAVI	1 year	78 ± 8	45 (45%)	Severe: 100	On univariate analysis, Troponin was associated with all-cause mortality (HR 5.41, 95% CI 2.01-14.58, p=0.001). Cardiovascular events defined as CV death, non-fatal MI, and stroke or TIA. On univariate analysis, Troponin was associated with CV events (HR 4.18, 95% CI 1.35–12.97, p=0.013).
Chin CW et al. 2014[63]	131	Asymptomatic moderate to severe AS (SALTIRE study)	10.6 years	67 ± 10	91 (70%)	N/A	Plasma cTnI concentration was associated with an increased risk of AVR or CV deaths in unadjusted analysis (HR 1.65 per two-fold increment in cTnI concentration; 95% CI, 1.15–2.38, P = 0.007) with minimal attenuation in the effect estimate after adjusting for age, sex, and ejection fraction. This association persisted after further adjustment for severity of aortic stenosis (HR 1.77; 95% CI, 1.22–2.35, P = 0.002) as well as the coronary artery and aortic valve calcium scores (HR 2.10; 95% CI, 1.22–3.61, P = 0.007).
Chorianopoulos E et al. 2014[73]	198	Patients undergoing transfemoral TAVI	12 months	81 ± 7.8	71 (36%)	Severe: 198	A late rise in cTnT levels beyond day three after TAVI was a significant predictor of 30-day all-cause mortality (P = 0.0233, HR 4.74; 95 % CI 1.3–17.4), whereas neither the baseline cTnT level nor the early change (absolute concentration change) were useful for the prediction of short-term outcome. In contrast, baseline cTnT values were strongly predictive for mortality at 1 year. Thus, patients with baseline cTnT levels above the median (29 pg/mL) had significantly higher mortality rates than those with cTnT levels below the median (p = 0.024) and this was significant on univariate analysis (HR 3.34, 95% CI 1.17- 9.54).
Dahou A et al. 2018[22]	98	Low-flow low-gradient AS (TOPAS study)	2.8 years	73.7 ± 9.8 years	74 (75%)	N/A	Those with hsTnT ≥15 ng/l had an increased risk of mortality compared with those with hsTnT <15 ng/l. 2-year mortality was higher in group C (41 ± 9%) than in group B (23 ± 7%) and group A (5 ± 4%) (p = 0.002). Having either BNP or Hs-TnT raised and having both BNP and hs-TnT raised were significantly associated with increased mortality (Group B: HR 5.0, 95% CI 1.68–21.39, p=0.0024) and (Group C: HR 6.85, 95% CI 2.37–28.98, p=0.0001), respectively. The effect of elevation of BNP and hsTnT on mortality was similar after adjustment for the presence of renal failure (HR: 5.13; 95% CI = 1.37 to 25.30; p = 0.01).
Ferrer-Sistach E et al. 2019[67]	58	High gradient ASAS	1 year ± 30 days	74.8 ± 8.4	33 (56.9%)	Severe: 58	Primary endpoint was a composite of CV death, new-onset symptoms, cardiac hospitalization, guideline-driven indication for valve replacement and CV death at 12m. It was associated with high sensitivity troponin T (hs-TnT) (P<0.001). In multivariable analysis, the primary endpoint only remained significantly independently associated with aortic regurgitation of ≥2 (P=0.01) and hs-TnT (P=0.007) (Table 2). ROC analysis revealed that the best cut-off value for hs-TnT was 10 ng/L. In patients with hs-TnT>10 ng/L, the risk of the primary outcome was ~10-fold higher than in patients with hs-TnT≤10 [HR, 9.62 (2.27–40.8); P=0.002].

Frank D et al. 2013[48]	107	Patients underoging TAVI	249 days (± 158)	81.63 ± 6.34	38.30%	N/A	On univariate analysis hsTnT above the upper quartile (>48.4 ng/mL) was associated with mortality (HR 6.06, 95% CI 2.51–14.64, P<0.001). A Cox multivariate regression analysis revealed that hsTnT (HR upper quartile 5.1 vs. lower quartiles, CI 2.06–12.54, p = 0.001) independently predict survival. An elevated hsTnT was associated with reduced EF (log rank test p = 0.018), systolic pulmonary hypertension N55 mm Hg (p = 0.001), and mild as well as moderate preprocedural aortic regurgitation (p = 0.012).
Kim JB et al. 2017[26]	112	Severe AS undergoing TAVR	679 days	84.0 ± 8.5	66 (59%)	Severe: 112	Of the biomarkers assayed, troponin wasn't found to associate with mortality. LVMI was associated with hs-TnI (r=0.26; P<0.01). hs-TnI also showed significant difference in survival based on predefined cutoff values of 6 ng/L.
Kofler M et al. 2017[74]	681	Severe symptomatic AS undergoing TAVR	434 days (IQR 148-783 days)	83 years (IQR: 79-86)	302 (44%)	Severe: 681	In regression analysis, hs-cTnT remained independently related to mortality during follow-up, as well as to 30-day mortality (odds ratio 2.715; 95% CI: 1.308 to 2.551). hs-cTnT was multiple associated with mortality in transfemoral (HR: 2.881; 95% CI: 1.841 to 4.509; p < 0.001) and transapical (HR: 1.954; 95% CI: 1.249 to 3.058; p = 0.009) TAVR. Hs-cTnT ≥35 ng/l was chosesn as optimal cutoff for prediction of mortality and after multivariate analysis was associated with all-cause mortality (HR 1.981, 95% CI 1.413–2.778, p<0.001).
Kohler WM et al. 2016[50]	259	AS undergoing TAVI	290 (Q1=88; Q3=529) days	83 ± 6.1	44.80%	N/A	Higher hsTNT levels (cut-off 46.1 pg/mL) was linked to increased TAVI mortality after both univariate (HR 2.94, 95% CI 1.83-4.75, p<0.001) and multivariate (HR 2.28, 95% CI 1.38-3.76, p=0.001) analysis. Analysis of Kaplan-Meier curves confirmed the significance of some of these death-associated variables. Higher baseline hsTNT could be linked to kidney disease (p=0.028), pulmonary hypertension (p<0.001), higher overall risk scores (all p ≤0.001), increased NTproBNP levels (p=0.004), and several VARC-2 criteria (e.g., cardiovascular death; p=0.016).
Koifman E et al. 2017[75]	474	Severe AS	1 year	83 ± 8	234 (49.4%)	N/A	Postprocedural cTn levels of >15 ULN weren't associated with all-cause mortality at 30 days (5% vs. 5.5%, p = 0.83) or at 1 year (19.5% vs. 21.6%, p = 0.65). There was good correlation between the cTn level and CKMB elevation with a Pearson correlation coefficient of r = 0.72 (p <0.001). The receiver operating characteristic curve reveals that the level of cTn that corresponds to 5 times the CKMB elevation is 75 times the ULN of cTn.
Lindman BR et al. 2018[52]	665	Patients undergoing surgical AVR	10.7 years	71 (63–77)	621 (93%)	Severe: 665	The highest HR for mortality were seen with higher levels of high-sensitivity cardiac troponin T, HE4, and GDF15. After standardized log2 transformation unadjusted hs-TnT was associated with mortality (HR: 1.34, 95% CI: 1.22-1.48, p<0.001). Hs-TnT was still associated with all-cause mortality after adjustment for Parsonnet score (HR 1.25, 95% CI 1.13-1.38, p<0.001) and EuroSCORE 2 (HR 1.27, 95% CI 1.15-1.40, p<0.001). The hazard associated with biomarker elevations for cardiovascular mortality was more pronounced than for all-cause mortality.
Lindman BR et al. 2015[53]	345	Patients with severe AS	1.9 ± 1.2 years	78 (11)	194 (66%)	Severe: 345	Both hs-TnT standardized log2 transformation and hs-TnT ≥24.5 (median) were found to be associated in univariate analysis with all-cause mortality (HR 1.39, 95% CI 1.16-1.66, p<0.001) and (HR 1.58, 95% CI 1.04-2.41, p=0.033), respectively. This association remained significant after log2 was adjusted for STS score (HR 1.26, 95% CI 1.03-1.53, p=0.022) and clinical factors (HR 1.20, 95% CI 0.98-1.46, p=0.08), and hs-TnT ≥24.5 was adjusted for STS score (HR 1.20, 95% CI 0.77-1.87, p=0.41) and clinical factors (HR 1.22, 95% CI 0.77-1.93, p=0.39).
Saito T et al. 2013[77]	60	Patients with AS undergoing AVR	922 ± 800	68.7 ± 9.6 years	30 (50%)	Severe: 60	Primary endpoint was the occurrence of MACE defined as events of heart failure, fatal arrhythmia, and all causes of death. Kaplan–Meier curve revealed a statistically significant difference in MACE rate among the groups (log-rank test, 2 = 13.0, p = 0.002). hs-TnT was found to be associated with MACE (HR 4.37, 95% CI 1.53–12.5, p=0.64). In a multivariate cox proportional hazard analysis with a model included age, sex, estimated glomerular filtration rate, hs-TnT tertile remained a significant factor to predict MACE (HR 3.71, 95% CI 1.16–11.9, p=0.03).

Sinning JM et al. 2016[76]	276	Patients with AS undergoing TAVI	1 year	80.9 ± 6.2	151 (54.7%)	N/A	In univariate analysis a chronic tropinin elevation >0.02 ng/mL at 3 months after TAVI was found to associate with mortality (HR 4.5, 95% CI 2.0-10.0, p<0.001). In multivariate Cox regression analysis with adjustment for independent univariate predictors of outcome, a chronic tropinin elevation >0.02 ng/mL at 3 months after TAVI (HR 4.4, 95% CI 2.0-9.7, p<0.001) were independently associated with outcome after one year.
Solberg OG et al. 2012[58]	136	Severe symptomatic AS	37 (1 – 54) months	74 ± 9	57%	Severe: 136	The unadjusted HR when comparing quartile 4 with quartile 1 – 3 for hsTnT was 9.89 (95% CI 2.90 – 33.75, p<0.001). Unadjusted log e transformation for hsTnT levels was also associated with mortality (HR 11.03, 95% CI 1.49 – 5.66, p=0.003). A combined model of NT-proBNP and hsTnT was also associated with mortality [Log e] HsTnT [Log e] NT-proBNP: unadjusted HR 1.96 (1.62 – 2.38), p< 0.001) which persisted after multivariable (stepwise) analysis (HR 2.16, 95% CI 1.65 – 2.81, p<0.001).
Stundl A et al. 2017[60]	461	Severe symptomatic AS	1 year	81.3 ± 6.4	234 (50.8%)	Severe: 461	Death at 30-days was 27 (5.9%) and at 1-year was 119 (25.8%). In univariable analysis, hs-TnT in ng/ml was not found to be associated with all-cause mortality (OR 1.332, 95% CI 0.946–1.876, p=0.101).

Table 5. Characteristics of including studies reporting Galectin-3

Author/Year	*N*	Population	Follow-up	Age (years)	Male (%)	AS severity (N)	Main findings
Agoston-Coldea L et al. 2018[62]	42	Severe AS	347 days (60-450 days)	73 ± 6	23 (54.8%)	Severe: 42	MACE was defined as sudden cardiac death, non-fatal myocardial infarction, ventricular arrhythmias, atrial arrhythmias, and hospitalization for heart failure. Average Galectin-3 level in those with no events was 15.2 (5.1-23.6) ng/mL and 17.7 (2.2-24.4) ng/mL in those with events. On univariate analysis Galectin-3 was associated with MACE (unadjusted OR 1.08 (0.95-1.82), p<0.01). This was insignificant on multivariate analysis (adjusted OR 0.97 (0.86-1.13), p=NS).
Baldenhofer G et al. 2014[78]	101	Severe AS undergoing transfemoral TAVI	1 year	78 ± 8	45 (45%)	Severe: 101	Kaplan–Meier analysis demonstrated a higher incidence of the VARC safety endpoint at 30 days when galectin-3 was above 17.8 ng/ml (41.7% vs. 13.8%, p = 0.002). Patients had a significantly better survival after TAVI within one year when galectin-3 levels were below 17.8 ng/ml (92.3% vs. 69.4%, p = 0.002). Cox regression analysis revealed significantly increased risk for reaching the 30-day VARC safety endpoint (HR: 3.36; 95% CI: 1.47–7.69; p = 0.004), of death (HR: 4.48; 95% CI: 1.56–12.91; p = 0.005), and of cardiovascular events (HR: 5.12; 95% CI:2.10–12.47; p = 0.001) for patients with elevated galectin-3. The significance was not lessened by taking into account possible confounders such as impaired renal function, gender, age, and NT-proBNP.
Bobrowska B et al. 2017[79]	80	Symptomatic degenerative AS	523 days	79 ± 8	36 (45%)	N/A	Gal-3 tended to predict all-cause mortality (Gal-3 >17.8 vs. Gal-3 <17.8 ng/mL; HR: 2.03 (95% CI 0.88–4.69), p = 0.09), which was abolished upon adjustment for eGFR (HR: 1.70 (0.61–4.73), p = 0.3). Galectin-3 per 1 SD was also associated with significance (HR 1.49, 95% CI 1.00–2.21, p=0.05), which didnt remain significant on adjustment for eGFR (HR 1.46, 95% CI 0.83–2.57, p=0.19). However, in post-BAV patients multivariate-adjusted pre-procedural Gal-3 was associated with worse survival (HR: 7.41 (1.52–36.1), p = 0.01) regardless of eGFR. Depressed eGFR as a strong determinant of circulating Gal-3 levels. Baseline Gal-3 correlated negatively with eGFR and positively with NT-proBNP.
Lindman BR et al. 2015[53]	345	Patients with severe AS	1.9 ± 1.2 years	78 ± 11	194 (66%)	Severe: 345	Galectin-3 above the median (≥3.66) and standardized log2 transformtion were both significantly associated with all-cause mortality (HR 1.86, 95% CI 1.21-2.84, p<0.001) and (HR 1.42, 95% CI 1.16-1.73, p<0.001), respectively. Median Galectin-3 did not remain significant after adjusting for STS (HR 1.20, 95% CI 0.77- 1.87, p=0.41) and clinical factors (HR 1.22, 95% CI 0.77-1.93, p=0.39). STandardized log2 transformation of Galectin-3 did not remain significant after adjusting for STS (HR 1.15, 95% CI 0.91- 1.45, p=0.25) or clinical factors (HR 1.21, 95% CI 0.96-1.53, p=0.10).
Rheude T et al. 2019[69]	439	Symptomatic severe AS undergoing TAVI	371 days [219-402]	81.0 (77.0-85.0)	240 (54.7%)	Severe: 439	Galectin-3 was dichotomized at ≥8.71 ng/mL into elevated and not elevated. On univariate analysis, elevated galectin-3 was associated with all-cause mortality (HR: 2.12, 95% CI: 1.12-4.05, p=0.022). This did not remain significant on multivariate analysis (HR: 1.14, 95% CI: 0.56-2.35, p=0.717). Composite of all-cause mortality or readmission for worsening heart failure after TAVI. Association of elevated Gal-3 with the primary endpoint was significant on univariate analysis (HR 2.26, 95% CI 1.42-3.59, p<0.001) and borderline significant on multivariate analysis (HR, 1.59, 95% CI 0.97-2.62, p=0.068).

Table 6. Characteristics of included studies reporting other biomarkers

Author/Year	*N*	Population	Biomarker	Follow-up	Age (years)	Male (%)	AS severity (N)	Main findings
Agoston-Coldea L et al. 2018[62]	42	Severe AS	CRP	347 days (60-450)	73 (6)	23 (54.8%)	Severe: 42	MACE was defined as sudden cardiac death, non-fatal myocardial infarction, ventricular arrhythmias, atrial arrhythmias, and hospitalization for heart failure. On logistic regression univariate analysis CRP was associated with MACE (OR 1.20, 95% CI 1.17-1.83, p=0.02). This did not remain significant on multivariate analysis (OR 1.06, 95% CI 0.94-1.14, p= NS).
Agoston-Coldea L et al. 2019[42]	52	Severe AS undergoing AVR	hs-CRP	386 (60 to 730) days	66 (7.5)	29 (55.7%)	Severe: 52	In Cox regression analysis, only reduced LAS (HR 1.33, 95% CI (1.01 to 1.74), p < 0.01) and LGE (HR11.3, 95% CI (1.82 to 70.0), p < 0.01) were independent predictors for MACEs. Kaplan–Meier curves for event-free survival showed a significantly higher rate of MACEs in patients with LGE (p < 0.01) and decreased LAS (p < 0.001) at CMR imaging. A stepwise multivariate Cox regression model was constructed, including age, 6MWD, E/E'ratio, LVEF, LAS and the presence of LGE. Only reduced LAS (HR 1.33 (95% CI 1.01–1.74; chi-square: 15.1, p < 0.001) and LGE (HR 11.3 (95% CI 1.82–70.2); chi-square: 24.3, p < 0.001) were independent predictors for the combined end-point.
Anand A et al. 2018[85]	104	Patients with moderate to severe AS	Cardiac myosin-binding protein (cMyC)	11 years (3882–4161) days	68.2 (9.8)	71 (68.3%)	Moderate: 52<br>Severe: 52	In Cox proportional hazards analysis, cMyC concentration was associated with an increased risk of all-cause mortality over the follow-up period after AVR as a time-varying covariate (HR 1.49 per log unit increase of cMyC, 95%CI 1.11 to 2.01, P=0.009). However, following adjustment for age, sex, AVmax or CT coronary calcium scores, statistical significance was lost.
Antonini-Canterin F et al. 2008[37]	64	Patients with AS (valve area 0.9 ± 0.3 cm2)	CA125	8 months (IQR 4.5–10)	76 ± 9	45.30%	Not reported	Composite end-point consisted of cardiac death, urgent aortic valve replacement, hospitalization for congestive heart failure. Both CA125 and BNP levels were significantly higher at baseline in the 18 patients who reached one of the pre-specified end-points during follow-up: 50.3 ± 58.4 vs. 18.9 ± 39.4 U/mL (p= 0.016) for CA125, and 981 ± 821 vs. 142 ± 158 pg/mL for BNP (pb0.001), respectively.
Auensen A et al. 2017[44]	442	Severe AS undergoing AVR evaluation	hs–CRP	3 years	74 ± 11	249 (56)	Severe: 442	Adjusted analysis showed that none of the studied biomarkers (NT–proBNP, hsTnT or hs–CRP) or any other covariates were associated with 3–year all–cause mortality following SAVR. MACE defined as all-cause death, TIA, stroke and MI. Adjusted analyses showed that hsTnT (HR, 1.51; 95% CI 1.11–2.05; P = 0.008) and left ventricular ejection fraction (HR 0.97; 95% CI 0.94–0.97, P = 0.043) was associated with MACE for operated patients during 1 year. NT–proBNP, hsTnT and hs–CRP had no independently prognostic value in relation to mortality following SAVR, hsTnT was associated with MACE following surgery.
Baldenhofer G et al. 2017[45]	100	Severe AS undergoing TAVI	MR-proADM<br>MR-proANP	1 year	78 ± 8	45	Severe: 100	On univariate analysis, MR-proADM (HR 3.34, 95% CI 1.08–10.35, p = 0.037), MR-proANP (HR 4.94, 95% CI 1.41–17.33, p = 0.013), NT-proBNP (HR 4.94, 95% CI 1.41–17.33, p = 0.013), CRP (HR 3.29, 95% CI 1.06–10.19, p = 0.039), Troponin (HR 5.41, 95% CI 2.01–14.58, p<0.001) were all significant with all-cause mortality. The combined biomarker models were also significant MR-proADM+Troponin+CRP (HR 5.41, 95% CI 2.01–14.58, p<0.001), MR-proANP+Troponin+CRP (HR 5.41, 95% CI 2.01–14.58, p<0.001), NT-proBNP+Troponin+CRP (HR 5.41, 95% CI 2.01–14.58, p<0.001) and MR-proADM+MR-proANP+NT-proBNP (HR 7.03, 95% CI 2.26–21.82, p<0.001).

Brynildsen J et al. 2019[86]	57	Moderate to severe AS referred for presurgical evaluation	Secretoneurin (SN)	3.5 (Q1–3 2.9–3.8) years (median)	77 (Q1–3 70–80)	26 (45.6%)	Not reported	Higher SN concentrations were associated with increased risk of mortality also after adjustment for established risk indices, biomarkers, and status regarding valvular surgery: hazard ratio per lnSN 15.13 (95% CI 1.05–219.00); p = .046. Patients with SN concentrations above the optimal cut-off (147 pmol/L) experienced ~7 times increased risk of mortality compared to patients below the optimal cut-off in adjusted models: HR 6.83 (1.74–26.81), p = .006. SN was higher in non-survivors than survivors: 156 (133–209) vs. 140 (116–155) pmol/L, p = .007. Higher SN caused increased risk of mortality after adjustment for established risk indices, biomarkers, and status regarding valvular surgery: HR per inSN 15.13 (95% CI 1.05–219.00); p = .046.
Capoulade R et al. 2015[87]	220	Mild-to-moderate AS	Lipoprotein (a)<br>OxPL-apoB	3.5 +/- 1.2 years	58 +/- 13	60	Not reported	After adjustment for age, sex, and baseline AS severity, patients in the top Lp(a) tertile (hazard ratio: 2.0; 95% CI: 1.1 to 3.7; p < 0.02) or the top OxPL-apoB tertile (hazard ratio: 1.9; 95% CI: 1.0 to 3.4; p < 0.04) had a significantly higher rate of clinical events. The secondary outcome was the composite of AVR or cardiac death. On multivariable analyses adjusted for age, sex, hypertension, smoking history, metabolic syndrome, systolic blood pressure, statin use, corrected LDL-C, apoB, creatinine, bicuspid aortic valve phenotype, aortic valve calcification score, baseline Vpeak, and valvuloarterial impedance, both top tertiles of Lp(a) (beta coefficient: 0.21 0.04; p < 0.01) or OxPL-apoB (beta coefficient: 0.21 0.04; p < 0.02) remained independent predictors of faster progression rate assessed by Vpeak.
Cho KI et al. 2016[64]	336	Severe calcific AS (newly diagnosed)	Neutrophil-to-lymphocyte ratio<br>hs-CRP	33 months	70.1 +/- 12.0 years	166 (49.4%)	Severe: 336	MACE was defined as a composite of all-cause mortality, cardiac death and non-fatal myocardial infarction during the follow-up period. NLR, was the independent prognostic factor most significantly associated with MACE (multivariable HR, 1.06; 95% CI, 1.04–1.09; p-value < 0.001, Simple: HR 1.07; 95% CI, 1.05-1.09; p-value<0.001. Simple hs-CRP, mg/L: HR 1.09, (1.04-1.14), p<0.001 and Simple NT-proBNP: HR 1.00, (1.00-1.00), p<0.001.
Dahl JS et al. 2012[88]	125	Severe AS undergoing AVR	Fibulin-1	3.8+/-1.5 years (median 4.0 years).	1st: 71 +/- 8<br>2nd: 72 +/-11<br>3rd: 74 +/-8	1st: 34 (81%)<br>2nd: 22 (52%)<br>3rd: 23 (56%)	Severe: 125	Cardiac mortality was significantly increased in patients with increased plasma fibulin-1 levels. In a univariable Cox regression analysis, fibulin-1 was associated with cardiac survival (HR 1.01, 95% CI 1.00-1.03, HRprSD 1.62, p=0.004). In a multivariable Cox model including the predefined variables of age, LA volume, and diabetes, fibulin-1 remained associated with cardiac mortality (HR 1.01, HRprSD 1.41, p=0.04).
Dahl JS et al. 2013[89]	124	Symptomatic severe AS planned for AVR	OPG	3.8 +/- 1.5 years (median 4.0 years)	Low: 67 ± 10<br>Mid: 73 ± 7<br>High: 77 ± 7	Low: 31 (76%)<br>Mid: 27 (64%)<br>High: 40 (49%)	Severe: 124	Univariable preoperative predictors of postoperative cardiac death: NT-proBNP (pmol/L) HR 1.00, 95% CI 0.99-1.01, adjusted HR 1.11, p=0.50. In a univariable Cox regression analysis OPG, age, EuroSCORE, gender, left ventricular mass index, left atrial volume index, s0avg, E/e0, and NT-proBNP were associated with a poor postoperative outcome, although in a stepwise regression model with forward selection of the aforementioned variables, only OPG was independently associated with a poor postoperative outcome. In a multivariable model adjusting for the predefined variables age, LV ejection fraction, NT-proBNP, and LA volume index, OPG was still associated with postoperative outcome (HRadjusted 1.64, p <0.001).

Ferrer-Sistach E et al. 2019[67]	58	High gradient ASAS	ST2	1 year +/- 30 days (primary endpoint)	74.8 ± 8.4	33 (56.9%)	Severe: 58	Primary endpoint was a composite of CV death, new-onset symptoms, cardiac hospitalization, guideline-driven indication for valve replacement and CV death at 12m. It was associated with aortic regurgitation of ≥2 (defined as more than mild) (P<0.001), left atrium indexed volume (LAVi) (P=0.001), NT-ProBNP (P<0.001), high sensitivity troponin T (hs-TnT) (P<0.001), diastolic dysfunction of ≥2 (P=0.03), and longitudinal strain (P=0.008). In patients with hs-TnT>10 ng/L, the risk of the primary outcome was ~10-fold higher than in patients with hs-TnT≤10 [HR, 9.62 (2.27–40.8); P=0.002]. In multivariable analysis, the primary endpoint only remained significantly independently associated with aortic regurgitation of ≥2 (P=0.01) and hs-TnT (P=0.007) (Table 2). ROC analysis revealed that the best cut-off value for hs-TnT was 10 ng/L.
Kim JB et al. 2017[26]	112	Severe AS undergoing TAVI	CRP<br>GDF-15<br>Cys-C (cystatin-C)	679 days (median)	84.0 ± 8.5	66 (59%)	Severe: 112	Among biomarkers, there was an age-related progressive increase in the biomarker levels, more prominent for GDF-15, BNP, and Cys-C. Of the biomarkers assayed, GDF-15 and CRP emerged as significantly associated with all-cause mortality on both univariate analysis (HR: 1.45, 95% CI: 1.17–1.82, p<0.001), (HR: 2.36, 95% CI: 1.61–3.44, p<0.001) and multivariate analyses (HR: 2.03, 95% CI: 1.40–3.00, p<0.001), (HR: 1.28, 95% CI: 1.00–1.63, p=0.05) respecitively. Furthermore, survival analysis based on previously defined cutoffs for GDF-15 (2000 pg/mL) and CRP (2 mg/L) showed significantly worse survivals in subgroups with elevated biomarker levels (GDF-15, log-rank P<0.0001; CRP, log-rank P=0.01).
Krau NC et al. 2015[51]	217	Patients with severe AS undergoing TAVI	GDF-15	349 days (106–660) (median)	81.8 ± 6.0 years	44.20%	Severe: 217	Median pre-procedural GDF15 values were 2256 pg/mL (1585–3082). High GDF15 levels were associated with numerous factors that could contribute to poor outcome. Analyses comparing the upper quartile with the lower three quartiles for biomarker levels revealed a HR of increased GDF15 for adverse outcome of 2.4 [95% confidence interval (CI) 1.5–3.9, P < 0.001]. Of note, in multiple analyses, elevated GDF15 levels were superior to NT-proBNP for predicting negative outcome (adjusted HR of GDF15 1.97, 95% CI 1.2–3.3; P = 0.009). Notably, a GDF15 level in the upper fourth quartile showed a significant association with reduced survival time after TAVI (P <0.001). Cox analyses revealed that increased GDF15 showed a HR for adverse outcome of 2.40 [95% CI 1.47–3.93, P <0.001]. In multiple Cox regression analysis, NT-proBNP did not remain significant when adjusted for GDF15.
Lindman BR et al. 2018[52]	665	Patients undergoing surgical AVR	Human epididymis protein (HE4)<br>Cancer antigen 125<br>GDF15<br>hs-CRP<br>Soluble ST2	10.7 years (median)	71 (63–77)	621	Severe: 665	The highest hazards for mortality were seen with higher levels of high-sensitivity cardiac troponin T, HE4, and GDF15. Standardized log2 transformed (unadjusted): hs-CTnT (HR: 1.34, 95% CI: 1.22-1.48, p<0.001), HE4 (HR: 1.86, 95% CI: 1.67-2.08, p<0.001), CA125 (HR: 1.27, 95% CI: 1.15-1.39, p<0.001), GDF15 (HR: 1.69, 95% CI: 1.52-1.88, p<0.001), NTproBNP (HR: 1.39, 95% CI: 1.24-1.56, p<0.001), hsCRP (HR: 1.28, 95% CI: 1.14-1.43, p<0.001) and ST2 (HR: 1.30, 95% CI: 1.17-1.44, p<0.001).

Lindman BR et al. 2015[53]	345	Patients with severe AS	GDF15<br>ST2<br>MPO<br>hsCRP<br>MCP-1	1.9 ± 1.2 years (mean)	78 (11)	194 (66%)	Severe: 345	After adjustment for STS risk score, a greater number of elevated biomarkers was associated with increased mortality (referent: 0 elevated): 1 elevated (HR 1.47, 95% CI 0.60 to 3.63, p=0.40), 2 elevated (HR 2.89, 95% CI 1.24 to 6.74, p=0.014) and 3 elevated (HR 4.59, 95% CI 1.97 to 10.71, p<0.001). HR are for no. biomarkers elevated, correlations for individual biomarkers are in supplementary table S1. When added to the STS score, the number of biomarkers elevated provided a category-free net reclassification improvement of 64% at 1 year (p<0.001).
Lutz M et al. 2017[90]	217	Severe symptomatic AS undergoing TAVI	Osteopontin (OPN)	349 days (median)	82 (78–86)	96 (44.2%)	Severe: 217	OPN levels in the highest quartile also showed a significant association with reduced survival time after TAVI (p=0.002). Preoperative OPN was only prognostic for survival within the first 6 months after TAVI. Within the subgroup of patients still alive after 6 months, baseline OPN levels are not related to further survival (p=0.32 log rank test). Cox analysis comparing upper quartile to the lower three quartiles of continuous variables. This analysis shows that increased OPN levels considered individually have a HR for adverse outcome of 2.15 (95% CI 1.3 to 3.5, p=0.002). In multiple Cox regression analysis, NTproBNP did not remain significant when adjusted for OPN. In contrast, the upper quartile of OPN itself revealed an HR of 2.06 (CI 1.2 to 3.5, p=0.005).
Hioki H et al. 2018[91]	1544	Severe symptomatic AS undergoing TAVI (OCEAN registry)	CRP<br>hs-CRP	2 years	Low CRP: 85 (82–88)<br>High CRP: 85 (82–88)<br>Low hs-CRP: 84 (81–87)<br>High hs-CRP: 85 (81–88)	454 (29.4%)	Severe: 1544	The Kaplan-Meier curve demonstrated that the incidence of 2-year all-cause death was significantly higher in patients with high CRP than those with low CRP (11.5% vs. 7.6%, log-rank P = 0.015). The impact of high CRP level on mortality was predominantly observed within the first 3-month of TAVI with an adjusted hazard ratio (HR) of 2.78 (95% confidence interval [CI], 1.30–5.95) in multivariate model. After 3-month of TAVI, the impact of high CRP on mortality was not observed anymore with an adjusted HR of 0.80 (95% CI; 0.47–1.36) (p for interaction = 0.008).
Hodges GW et al. 2016[92]	1503	Patients with mild-moderate AS	suPAR	4.3 years	68 ± 10	61%	Not reported	The multivariate adjusted hazard ratio (HR) (95% confidence interval [CI]) per unit log2 ng/ml increase in suPAR was 2.0 (1.4-2.9, p<0.001) for all-cause mortality. Univariate was 2.5 (2.0-3.1, p<0.001). The multivariate adjusted hazard ratio (HR) (95% confidence interval [CI]) per unit log2 ng/ml increase in suPAR was: 2.0 (1.2-3.3, p=0.007) for cardiovascular mortality. Univariate was 2.3 (1.7-3.3, p<0.001). Cox regression analysis was performed to evaluate associations between suPAR and the composite end-points of ischemic cardiovascular events (ICE), aortic valve events (AVE), cardiovascular and all-cause mortality. The multivariate adjusted hazard ratio (HR) (95% confidence interval [CI]) per unit log2 ng/ml increase in suPAR was: 1.5 (1.2-1.9, p=0.002) for ICE; 1.2 (0.9-1.5, p=0.071) for AVE.
Husser O et al. 2017[93]	422	Patients undergoing TAVI for severe AS	CA125	59 (range, 34-107) weeks (median)	79 ± 6	200 (47%)	Severe: 422	Elevated CA125 (> 30 U/mL) was present in 26% (110 of 422) of the patients and was associated with a significantly higher rate (47% [52 of 110], vs 20% [63 of 312]; P < .001) and risk of mortality (HR 2.76; 95%CI, 1.91-3.99; P < .001). In the multivariable analysis, CA125 (> 30 U/mL) remained an independent predictor of mortality (hazard ratio [HR], 2.16; 95% confidence interval [95%CI], 1.48-3.15; P < .001) and improved the predictive capability of the model (C-statistic: 0.736 vs 0.731) and the net reclassification index (51% 95%CI, 33-73) with an integrated discriminative improvement of 3.5% (95%CI, 0.5-8.4). No interaction was found between elevated CA125 and EuroSCORE but a combination of CA125 and the EuroSCORE significantly improved risk prediction.

Husser O et al. 2013[94]	228	Patients undergoing TAVI	CA125	183 days (IQR 63-365) and 144 days (IQR 56-365) (median)	79 ± 6	124 (54)	Not reported	In a multivariable analysis adjusted for logistic EuroSCORE, NYHA class III/IV, and device success, baseline values of CA125 (M2 vs. M1) independently predicted death (hazard ratio [HR]: 2.18; 95% confidence interval [CI]: 1.11 to 4.26; p 0.023). An approximate 3-fold increase in the rate of death and MACE was observed in patients with elevated baseline CA125 (M2 5.2 vs. M1 1.6 per 10 persons-year and M2 8.3 vs. M1 3.3, respectively; p for both 0.001) and for CA125 >30 U/ml versus CA125 ≤30: 6.1 versus 2.3 per 10 persons-year and 9.5 versus 4.2 per 10 personsyear, respectively; p for both 0.001). The endpoints of this study were all-cause death and major adverse cardiac events (MACE) defined as a composite of all-cause death, acute HF requiring admission, myocardial infarction, and stroke, whichever occurred first during follow-up. In a multivariable analysis adjusted for logistic EuroSCORE, NYHA class III/IV, and device success, baseline values of CA125 (M2 vs. M1) independently predicted MACE (HR: 1.77; 95% CI: 1.05 to 2.98; p 0.031).
Koifman E et al. 2017[75]	474	Severe AS	CKMB	1 year	CKMB ≤ 5: 83 ± 8<br>CKMB >5: 82 ± 9<br>cTn ≤15: 83 ± 8<br>cTn >15: 83 ± 8	CKMB ≤ 5: 206 (49%)<br>CKMB >5: 15 (41%)<br>cTn ≤15: 70 (64%)<br>cTn >15: 164 (45%)	Not reported	CKMB elevation of >5 ULN had more than triple the mortality rate at all-cause 30 days as patients with lower CKMB levels (14% vs. 4%, p <0.001) and double the mortality rate at 1 year (39% vs. 19%, p = 0.009). Postprocedural cTn levels of >15 ULN were not associated with all-cause mortality at 30 days (5% vs. 5.5%, p = 0.83) or at 1 year (19.5% vs. 21.6%, p = 0.65). CKMB elevation remained statistically significant after multivariate adjustment with approximately double the risk for all-cause mortality at 1 year (hazard ratio = 2.02, 95% confidence interval = 3.88 to 1.05, p = 0.035). Echocardiographic and procedural characteristics were also assessed. There was good correlation between the cTn level and CKMB elevation with a Pearson correlation coefficient of r = 0.72 (p <0.001). However, the receiver operating characteristic curve reveals that the level of cTn that corresponds to 5 times the CKMB elevation is 75 times the ULN of cTn.
Marchandot B et al. 2019[95]	106	Patients with severe AS (high or intermediate surgical risk) undergoing TAVR	Reactive oxygen species (ROS) via plasmatic superoxide anion (SA)	30 days	81 +/- 8	48 (45%)	Severe: 106	Composite endpoint of all-cause mortality and/or stroke and/or pacemaker implantation and/or significant paravalvular aortic regurgitation ≥ mild at 30 days did not differ significantly according to SA baseline values (p=0.055). Increased baseline SA (>75 percentile) was continuously associated with higher post procedural SA values 10 minutes after valve expansion (p<0.001), at 3 days (p<0.0001) and 30 days (p<0.001). Higher baseline SA was linked to higher inflammatory response assessed by higher CRP values at Day-1 and Day-3.
Muessig JM et al. 2019[96]	208	Severe symptomatic AS undergoing TAVI	Insulin like growth factor binding protein 2 (IGFBP-2)	278 days (259–297) (mean)	80.85 ± 0.49	N/A	Severe: 208	IGFBP-2 plasma concentration was found to be associated with increased 1-year mortality (changes per ng/ml IGFBP-2 concentration; HR 1.002 95% (1.002 (1.001–1.003); p b 0.001). One-month mortality was significantly higher in those patients with IGFBP-2 plasma levels above 275 ng/ml (3% vs. 11%, p = 0.05). Also after one year patients with IGFBP-2 levels above the optimal cut-off evidenced a significantly worsened outcome (18.2% vs. 46.2% mortality; p b 0.001).

Parenica J et al. 2012[32]	42	High-risk patients allocated to TAVI or SAVR	Creatinine, Cystatin C, ADMA Nitrite/nitrate, 8-OHdG, FRAP MDA, Cysteine, Homocysteine, Cysteinyl-Glycine, Glutathione, MMP-2 and 9, TIMP-1	1 year	82 (75; 89)	13 (31%)	Severe: 42	Malondialdehyde served as the best predictor of a combined endpoint at 1 year with AUC (ROC analysis) = 0.872 for TAVI group, resp. 0.765 (p<0.05) for both TAVI and SAVR groups. Increased levels of MDA, matrix metalloproteinase 2, tissue inhibitor of metalloproteinase (TIMP1), ferritin-reducing ability of plasma, homocysteine, cysteine and 8-hydroxy-2-deoxyguanosine were all predictors of the occurrence of combined safety endpoints at 30 days (AUC 0.750–0.948; p<0.05 for all). The addition of MDA to a currently used clinical model (EuroSCORE) significantly improved prediction of a combined safety endpoint at 30 days and a combined endpoint (0–365 days) by the net reclassification improvement (NRI) and the integrated discrimination improvement (IDI) (p<0.05). Cystatin C, glutathione, cysteinylglycine, asymmetric dimethylarginine, nitrite/nitrate and MMP9 did not prove to be significant.
Ramchand J et al. 2020[97]	127	Patients with AS	Plasma ACE2	5 years (IQR 0.19 to 5 years)	75 +/- 11	81 (64%)	Mild: 25% Moderate: 24% Severe: 51%	Patients with plasma ACE2 activity above 41.2pmol/ml/min this threshold had a significantly increased likelihood of all-cause mortality compared with those without activity (HR: 3.86; 95% CI: 1.85 to 8.06; p < 0.001). All-cause mortality was highest in those with the combination of elevated plasma ACE2 activity and BNP clinical activation (HR: 13.78; 95% CI: 3.97 to 47.8; p < 0.001). Elevated plasma ACE2 activity was an independent predictor of all-cause mortality after adjustment for relevant clinical, imaging, and biochemical parameters (HR: 2.28; 95% CI: 1.03 to 5.06; p = 0.042), including brain natriuretic peptide activation (integrated discrimination improvement: 0.08; p < 0.001). Elevated plasma ACE2 activity was an independent predictor of all-cause mortality after adjustment for relevant clinical, imaging, and biochemical parameters (HR: 2.28; 95% CI: 1.03 to 5.06; p = 0.042), including BNP activation (integrated discrimination improvement: 0.08; p < 0.001). Cox multivariable regression analysis demonstrated that elevated plasma ACE2 activity (HR: 2.28; 95% CI: 1.03 to 5.06; p =0.042) was an independent predictor of mortality in addition to age (HR: 1.07; 95% CI: 1.01 to 1.13; p = 0.015), a history of heart failure (HR: 3.07: 95% CI: 1.39 to 6.77; p = 0.006), and a history of IHD (HR: 2.96; 95% CI: 1.29 to 6.82; p = 0.011).
Rheude T et al. 2019[69]	439	Symptomatic severe AS undergoing TAVI	CA125	371 days [219-402] (median)	81.0 (77.0-85.0)	240 (54.7%)	Severe: 439	Elevated CA125: univariate (HR: 3.39, 95% CI: 1.55-47.43, p=.002), multivariate (HR: 2.25, 95% CI: 0.96-5.29, p=.062). Composite of all-cause mortality or readmission for worsening heart failure after TAVI. Association of elevated Gal-3 with the primary endpoint was borderline significant (HR, 1.59; P=.068). CA125 was associated with a higher risk of the primary endpoint (25.4% vs 6.6%, HR, 4.20; P<.001). After multivariable adjustment, elevated CA125 (HR, 2.83; P=.001) remained independently associated with the primary endpoint.
Rheude T et al. 2018[70]	363	Severe AS undergoing transfemoral TAVI	CA125	1 year	81 ± 6	197 (54%)	Severe: 363	All-cause mortality (13% [24 of 188] vs 2% [3 of 175]; p <0.001), cardiac death (9% [16 of 188] vs 2% [3 of 175]; p = 0.004), and readmission for CHF (19% [36 of 188] vs 2% [4 of 175]; p <0.001) were more frequent in case of elevated baseline CA125 levels. Primary end point was all-cause death or readmission for worsening congestive heart failure within 1 year after TAVI. After multivariable adjustment, elevated CA125 was independently associated with the primary end point (HR 5.26; 95% CI [2.13 to 13.00]; p <0.001).

Røsjø H et al. 2014[56]	57	Moderate to severe AS	miR-210<br>miR-22	1287 days (median)	75 ± 1	26 (46%)	Not reported	High NT-proBNP and miR-210 levels, together with history of hypertension and increasing eccentric LV hypertrophy based on calculation of relative wall thickness, were associated with mortality in multivariate Cox proportional hazard regression analysis. The prognostic accuracy of miR-210 for all-cause mortality was comparable to the accuracy of NT-proBNP levels: AUC = 0.64 (95% CI 0.50–0.76) vs. AUC = 0.67 (0.53–0.79), respectively, p = 0.83.miR-210 levels were higher in AS patients compared to the control subjects: 3.0 ± 0.2 vs. 1.0 ±0.5. miR-22 levels (Cq 27–30), which is another miRNA biomarker candidate, were not increased in the AS patients. miR-210 levels were correlated with miR-22 levels in the AS patients (r = 0.46, p<0.001), but not in the control subjects.
Schmid J et al. 2017[98]	74	Patients with severe AS undergoing TAVI	ST2<br>CK-MB	2.6 ± 1.3 years (mean)	83 ± 5.3 years	26 (35%)	Severe: 74	Patients with ST2 concentrations above the cut-off >49ng/mL were significantly more likely to die during follow up than patients below this threshold (one- and two-year mortality 40% and 60% vs. 7% and 15.8%, respectively; both p<0.001 by log-rank test). In univariate Cox regression analyses, ST2, STS score, and RA area significantly predicted all-cause mortality (ld ST2: HR = 3.21 (95% CI 1.42–7.24), p = 0.005; ST2 N 95%ile: HR = 3.84 (95% CI 1.64–8.96), p = 0.002; STS score: HR = 1.14 (95% CI 1.04–1.26), p = 0.008; RA area: HR = 1.07 (95% CI 1.02–1.12), p = 0.011). In a multivariate Cox regression model both ST2 and STS score significantly predicted mortality (ld ST2: HR = 2.92 (95% CI 1.10–7.75), p = 0.031; STS score: HR = 1.15 (95% CI 1.02–1.29), p = 0.026). ST2 predicted major adverse cardiovascular events (MACE, p = 0.046). ST2 also predicted the secondary endpoint one-year MACE in univariate Cox regression analysis (ld ST2: HR = 2.39 (95% CI 1.02–5.60), p = 0.046) and a multivariate Cox regression model containing ST2 and STS score (ld ST2: HR = 2.35 (95% CI 1.02–5.44), p = 0.045).
Sinning JM et al. 2015[57]	310	Patients with AS undergoing TAVR	hsCRP<br>GDF-15<br>IL-6<br>IL-8	1 year	83.0 (77.0-86.0)	165 (53.4%)	Not reported	The EuroSCORE II and GDF-15 had the strongest predictive value for 1-year mortality (EuroSCORE II, area under the curve (AUC) 0.711; GDF-15, AUC 0.686) and for the composite endpoint (EuroSCORE II, AUC 0.690; GDF-15, AUC 0.682). The prediction of 1-year death for all biomarkers was as followed: GDF-15: HR 2.4 (1.8-3.3), p<0.001, IL-8: HR 2.2 (1.5-3.1), p<0.001, NT-proBNP:HR 1.4 (1.2-1.7), p<0.001, hsCRP: HR 1.2 (1.0-1.4), p=0.012, IL-6: 1.1 (0.9-1.4), p=0.39.
Sinning JM et al. 2016[76]	276	Undergoing TAVI	CK-MB	1 year	80.9 +/- 6.2	151 (54.7%)	Not reported	In multivariate Cox regression analysis with adjustment for independent univariate predictors of outcome, an elevated troponin after TAVI at follow-up (HR 4.5, 95% CI: 2.0-10.0; p<0.001) were independently associated with outcome after one year. In 13.5% of the patients, baseline troponin was elevated above the URL (>0.10 ng/mL). From day 3 on, non-survivors after TAVI had significantly higher troponin levels compared to survivors (p<0.001). Non-survivors also showed elevated troponin levels at follow-up after three (p<0.001) and six months (p<0.001).
Steinmetz M et al. 2019[99]	155	Patients undergoing TAVR	Leucocyte telomere length	1 year	80.53 +/- 5.87	76 (49.0%)	Severe: 155	30-day and 1-year survival were not significantly different between the 3 groups with short, intermediate and long telomere length (30-days: 1st tertile 98.1%, 2nd tertile 98.1%, 3rd tertile 98.0%; 1-year: 1st tertile 90.4%, 2nd tertile 76.9%, and 3rd tertile 92.2%; all p>0.05). Kaplan-Meyer survival curves of TAVR patients did not show a significant correlation between relative telomere length and mortality (1st tertile: shortest telomeres, 2nd tertile: median telomeres, 3rd tertile: longest telomeres). Long telomere length was significantly associated with a reduced left ventricular ejection fraction at baseline (LVEF; 1st tertile: 58.6%; 2nd tertile: 58.3%; 3rd tertile: 49.9%; p less than or equal to 0.05 vs. 1st and 2nd tertile).

Stundl A et al. 2018[100]	683	Patients who underwent TAVI	hs-CRP	1 year	80.8 ± 6.0	334 (48.9%)	Severe: 683	Non-survivors at one year had higher risk scores and increased median biomarker levels. Logistic EuroSCORE in combination with hs-CRP had the highest predictive value for 30-day (AUC 0.740 [95% CI: 0.667-0.812], p=0.1117) and one-year mortality (AUC 0.631 [95% CI: 0.569-0.693], p=0.0403). In multivariate regression analysis, logistic EuroSCORE in combination with hs-CRP showed the strongest association with one-year mortality. Combinations of increasing medians of logistic EuroSCORE results and hs-CRP levels allowed the stratification of the TAVI patients into subgroups with one-year mortality rates ranging from 6.6% up to 18.2%. hs-CRP alongside the logistic EuroSCORE was an independent predictor of one-year all-cause mortality in TAVI patients.
Stundl A et al. 2017[60]	461	Patients with severe symptomatic AS undergoing TAVI	ST2	1 year	81.3 ± 6.4	234 (50.8%)	Severe: 461	Patients with ST2 levels >29.0 ng/ml showed both significantly increased 30-day mortality (9.7% vs 4.6%, p = 0.043) and 1-year mortality (38.1% vs 21.8%, p = 0.001) compared with patients with ST2 levels ≤29.0 ng/ml. After additional adjustment for NT-proBNP, ST2 remained an independent predictor of mortality (HR 1.712 [1.266 to 2.315], p <0.001 vs HR 1.295 [1.121 to 1.497], p <0.001). Patients with an ST2 >29.0 ng/ml (n = 113, 24.5%) had higher clinical risk scores, and showed more often underlying associated comorbidities.
Szekely Y et al. 2019[101]	1029	Severe symptomatic AS	Red blood cell distribution	up to 7.5 years	83.1 +/- 6.3 years	RDW <15.5%: 41% >15.5%: 49%	Severe: 1029	There were non-significant higher rates of in-hospital mortality and of 30-day mortality among the elevated RDW group. There was a significantly higher 1-year mortality rate [17% vs. 6%, HR = 2.18, 95% CI 1.37–3.47, p = 0.001] and 5-year mortality rate (38% vs. 20%, HR = 1.9, 95% CI 1.45–2.49, p < 0.001) among patients with elevated RDW. RDW (15.5% cutoff) univariable HR 2.14 (1.7–2.7) p<0.001, multivariable HR 1.98 (1.54–2.56) p<0.001.
Ueland T et al. 2011[102]	136	Severe symptomatic AS evaluated for AVR	OPG split into quartiles	37 months (1–54 months)	74 ± 10	55%	Severe: 136	In a multivariable forced Cox regression, diabetes, NT-proBNP and OPG were found to be significantly associated with all-cause mortality with the highest Wald value for OPG. A significant association between OPG and mortality was observed regardless of AVR [AVR 2.78 (1.53–5.05) P = 0.001; non-AVR 6.55 (2.09–20.55) P = 0.001]. Stepwise multivariable analysis as conducted earlier showed that OPG remained a significant predictor of all-cause mortality in both subgroups [AVR 2.75 (1.43–5.27) P = 0.002; non-AVR 4.86 (1.11–21.27) P = 0.036].
Wernly B et al. 2017[36]	274	Symptomatic severe AS	ST2	1 year	80 ± 0.5 years	125	Severe: 274	In a Cox regression analysis, ST2 plasma concentration was associated with increased mortality (HR 1.00006 95% (1.00004-1.00009); p<0.001). A cut-off by means of the Youden-Index was calculated (10070.27 pg/ml) and patients were retrospectively divided into those above (31.3%) and those below (68.7%) this value. Patients with a ST2 concentration above the cut-off of 10070.27 pg/ml showed a significantly worse outcome after one year (49.2% vs. 23.2%; OR 3.21 95%CI (1.70-6.04); p<0.001). After correction for confounders in a multivariate Cox regression analysis, ST2 (1.0002 95%CI (1.0001-1.0003); p=0.001) concentration remained associated with mortality.
